# Assessment of Anti-Alzheimer Pursuit of Jambolan Fruit Extract and/or Choline against AlCl_3_ Toxicity in Rats

**DOI:** 10.3390/toxics11060509

**Published:** 2023-06-06

**Authors:** Zeinab Abdel Salam Hawash, Ensaf M. Yassien, Badriyah S. Alotaibi, Amira M. El-Moslemany, Mustafa Shukry

**Affiliations:** 1 Nutrition and Food Science Department, Faculty of Home Economic, Al-Azhar University, Tanta 31732, Egypt; 2 Department of Pharmaceutical Sciences, College of Pharmacy, Princess Nourah bint Abdulrahman University, P.O. Box 84428, Riyadh 11671, Saudi Arabia; 3 Physiology Department, Faculty of Veterinary Medicine, Kafrelsheikh University, Kafrelsheikh 33516, Egypt

**Keywords:** jambolan, choline, aluminium chloride, brain functions, antioxidant enzymes

## Abstract

Jambolan fruit extract and choline were investigated for Aluminum tri chloride (AlCl_3_)-induced Alzheimer’s disease in rats. Thirty-six male “Sprague Dawley” rats weighing (150 ± 10 g) were allocated into six groups; the first group was fed a baseline diet and served as a negative control. Alzheimer’s disease (AD) was induced in Group 2 rats by oral administration of AlCl_3_ (17 mg/kg body weight) dissolved in distilled water (served as a positive control). Rats in Group 3 were orally supplemented concomitantly with both 500 mg/kg BW of an ethanolic extract of jambolan fruit once daily for 28 days and AlCl_3_ (17 mg/kg body weight). Group 4: Rivastigmine (RIVA) aqueous infusion (0.3 mg/kg BW/day) was given orally to rats as a reference drug concomitantly with oral supplementation of AlCl_3_ (17 mg/kg body weight) for 28 days. Group 5 rats were orally treated with choline (1.1 g/kg) concomitantly with oral supplementation of AlCl_3_ (17 mg/kg body weight). Group 6 was given 500 mg/kg of jambolan fruit ethanolic extract and 1.1 g/kg of choline orally to test for additive effects concurrently with oral supplementation of AlCl_3_ (17 mg/kg bw) for 28 days. Body weight gain, feed intake, feed efficiency ratio, and relative brain, liver, kidney, and spleen weight were calculated after the trial. Brain tissue assessment was analyzed for antioxidant/oxidant markers, biochemical analysis in blood serum, a phenolic compound in Jambolan fruits extracted by high-performance liquid chromatography (HPLC), and histopathology of the brain. The results showed that Jambolan fruit extract and choline chloride improved brain functions, histopathology, and antioxidant enzyme activity compared with the positive group. In conclusion, administering jambolan fruit extract and choline can lower the toxic impacts of aluminum chloride on the brain.

## 1. Introduction

The brain, a massive mass of nerve cells housed safely in the skull, serves as the central nervous system’s command center. The cerebrum, brainstem, and cerebellum are its three primary components. The brain regulates the body’s cognitive processes, such as taking in sensory data and figuring out how to use it. It’s a jelly-like tissue mass comprising 86 billion nerve cells and weighing around 1.4 kg [1]. Neurodegenerative illnesses, such as Alzheimer’s and neuropsychiatric disorders, include the larger category of human brain disorders. Although there is no cure, disc illnesses can be managed with medication, surgical procedures, and physical therapy [2].

Alzheimer’s disease (AD) is a form of dementia that causes gradual memory loss and other cognitive impairments. It’s the most common type of dementia in the elderly and a substantial financial and emotional drain on families and communities. People with AD are projected to reach 131.5 million by 2050 [3]. Neuritic plaques and neurofibrillary tangles from amyloid-peptide A buildup in the medial temporal lobe and neocortical brain regions is a hallmark of AD, a neurodegenerative disorder [4]. No cure exists for this ailment. Emotionally and financially, AD treatment and care are costly. Drugs for AD are expensive and only address symptoms. The cause of sporadic AD—more than 95% of cases—is unknown. It’s hard to avoid sporadic AD and identify risk factors because its etiology is unknown. A new study found that healthy lifestyle habits and comorbidity management may lessen dementia risk, even when avoiding or preventing modifiable risk factors may not entirely prevent the condition [5]. Suddenly appearing in adults, multiple factors, including genetics, lifestyle, and environment, all play a role in AD development and progression. Age, male gender, smoking, obesity, diabetes, hypertension, and cardiovascular disease are all risk factors for AD. With an aging population, therapeutic strategies that aim to delay or prevent cognitive decline by reducing modifiable risk factors are gaining popularity [6]. Smoking and alcohol use, as well as concurrent medical disorders such as cerebrovascular disease and depression, are also hotly contested contributors to dementia. Incidentally, Edwards et al. [5] examined the impact of changes to the cerebrovascular system, such as ischemia, and their possible role in the onset of AD. The epidemiological evidence pointing to stress as a significant contributor to depression and its involvement in triggering cognitive decline is striking. In the same vein, Amtul et al. [7] remark on the relationship between high cortisol levels and an increased risk of cognitive decline.

The disease manifests itself in the form of gradual cognitive and behavioral decline. Dementia is the most frequent form of Alzheimer’s disease, and it is anticipated that by 2050 there will be 152 million cases worldwide. Currently, this number stands at around 50 million people [8]. The hallmark of AD, as shown by neuropathological analysis, is the presence of neurofibrillary tangles composed of amyloid-protein (A) and hyperphosphorylated tau protein in the brain. When gathered in one place, they cause memory loss and neurological damage [9]. Sixty percent to eighty percent of dementia cases in the senior population are attributable to AD, making it one of the major global health issues of the century [10]. The cumulative effects of oxidative stress and inflammation may contribute to the loss of motor and cognitive abilities in aging humans and animals [11].

Cognitive decline with age and Alzheimer’s disease is tough on the brain’s glutamatergic pyramidal neurons [12]. Donepezil, rivastigmine, galantamine, and memantine form the basis of the current symptomatic treatment for mild-to-moderate AD patients. These medications reduce the severity of AD’s clinical symptoms but come with risks and limited efficacy [13]. The hunt for novel medicines of natural origin that protect against or prevent the onset, progression, or worsening of this neurodegenerative illness is in great demand. Drugs like these might lessen the adverse effects of currently used medications and promote healthy aging [14].

There is a significant unmet need to discover novel medications derived from natural sources that can prevent, delay, or reverse the worsening and progression of this neurodegenerative illness. This class of drugs can potentially lessen the adverse effects of currently used pharmaceuticals in clinical settings and promote healthy aging [15]. Acetylcholine, a neurotransmitter crucial for learning, memory, muscular control, and mood, is synthesized from the vital food choline [16].

Vitamins, minerals, fiber, and other bioactive components in fruits and vegetables are all vital to human health [17]. The antioxidant capacity and possible therapeutic effects on human health of phenol compounds among these bioactive chemicals have garnered significant interest [18]. The jambolan (*Syzygium cumini*) tree is a considerable evergreen in tropical and subtropical locations and is abundant in phenolic compounds. Other common names for this tree include jamun, jambul, black plum, and Indian blackberry [19]. Jambolan is a substantial bioactive phenolic chemical phenol source and may positively affect human health. Different portions of the jambolan plant contain varying amounts of phenolic chemicals, including phenolic acids, flavonoids (particularly anthocyanins, flavonols, flavanols, and flavanonols), and tannins. The skin of the jambolan fruit is rich in anthocyanins (such as delphinidin, petunidin, and malvidin in glycosylated forms). However, phenolic acids (such as gallic and ellagic acids) and tannins predominate in the pulp (mostly ellagitannins). The jambolan fruit has also been known to contain many additional chemicals. The seeds of the jambolan tree are reported to contain ellagic acid, gallic acid, and quercetin, while the leaves contain flavonoids such as quercetin, myricetin, and flavonol glycosides [20].

Inhibiting the generation of free radicals, polyphenols function as potent antioxidants, lowering the risk of ischemic, Parkinsonian, and Alzheimer’s diseases and other degenerative conditions caused by oxidative stress [21]. Polyphenols’ ability to boost brain health can be traced back to their ability to interact with neuronal and glial signaling pathways, thereby decreasing neurotoxin-mediated neuronal damage and loss, neuroinflammation, reactive oxygen species (ROS) production, and the accumulation of neuropathological markers like amyloid-b (Ab) and Tau protein [22]. Gallic acid and other polyphenol antioxidants have reversed age-related learning and memory decline in rats. Animal models of neurodegenerative disorders have shown that GA’s neuroprotective effects come from its involvement in antioxidant and inflammatory pathways [23].

Aluminum is a well-known environmental toxicant that has been shown to negatively impact brain development and morphology in several studies [24]. An increased aluminum concentration in the body has been linked to exposure to various everyday household items, including cooking utensils, food additives, drinking water, and medicine (antacids) [25]. Employees in the aluminum industry, in industries that use aluminum, and in welding significantly increase their exposure to the metal and/or its compounds due to the nature of their jobs [26]. In addition, prior studies and experiments have shown that aluminum poses a risk to the development of embryos in animal models and can be neurotoxic in humans and animals [27]. Research has also demonstrated that aluminum speeds up the degenerative process in AD by interacting with the cholinergic system in such a way that it modifies the function of cholinergic projections and also intensifies inflammation [28]. Aluminum promotes the formation of free radicals in the brain, which may result in neurodegenerative processes similar to those seen in Alzheimer’s disease [29]. In addition, it can securely connect to metal-binding amino acids (including histidine, tyrosine, and arginine) or phosphorylated amino acids, acting as a cross-linker due to its high positive charges and comparatively small ionic radius compared with other metal ions. Protein oligomerization and conformational changes induced by aluminum binding have been linked to the inhibition of protease-mediated protein breakdown and, by extension, to amyloid plaque development [30].

Clinical trials have examined many therapeutic approaches for decades, yet the present treatments are symptomatic [31]. Many attempts at treatment use lipid nutrients, “such as omega-3 polyunsaturated fatty acids [32]. The blood-brain barrier protects the CNS” from potential neurotoxins, toxic organisms, and chemical substances in the blood, but that also limits its accessibility to many therapeutic drug molecules [33].

Consuming foods rich in phenolic chemicals and their metabolites has been linked to numerous health benefits, including significant protection against age-related illnesses [34]. Gallic acid’s antioxidant, antibacterial, anti-inflammatory, anticarcinogenic, cardioprotective, gastroprotective, and neuroprotective capabilities have all been shown in multiple scientific studies [35]. Therefore, this study aimed to examine the effectiveness of jambolan fruit extract and choline in protecting rats from developing Alzheimer’s disease after exposure to aluminum trichloride.

## 2. Materials and Methods

### 2.1. Plant Material and Animals

The *Syzygium cumini* (jambolan) fruits used in this study were purchased from a local farmer in Tanta, Egypt. From the animal colony at Helwan Farm, Vaccine and Immunity Organization, Cairo, Egypt, thirty male albino rats weighing 150 ± 10 g were obtained. The animals included in the study were in good health and had been acclimated to the laboratory environment for at least a week before the experiment began. During this time, the rats lived in a quiet environment with natural ventilation and a 12:12-h light-dark cycle in plastic cages with galvanized iron filter tops. A standard rodent diet was used [36], and free access to water was provided during the experiment. The rules for the care and use of the animals used in the experiments were authorized by the Research Ethical Committee of the Faculty of Veterinary Medicine at the University of Kafr El-Sheikh in Egypt.

### 2.2. Chemicals and Kits

Casein, vitamins, minerals, cellulose, choline chloride, DL-methionine, and other necessary substances were purchased from El-Gomhoreya Company, Cairo, Egypt. The biochemical determination kits were obtained from Gama Trade Company, Cairo, Egypt. In the Al-Gharbia Governorate of Egypt, Tanta City was where corn starch and corn oil were purchased. Sigma Chemical Company provided aluminum chloride anhydrous (AlCl_3_) with a molecular weight of 133.34. NOVARTIS Pharmaceuticals in Cairo, Egypt, supplied the traditional medicine (Exelon-Rivastigmine—1.5 mg).

### 2.3. Extract Preparation

The fruit pulp was scooped out by hand, rinsed thoroughly under running water to eliminate debris, and then freeze-dried (Christ Beta 2e8 LD plus, Germany). The dehydrated pulp was kept at 20 °C for later testing. Twenty grams of freeze-dried jambolan pulp were combined with three hundred milliliters of 70% ethanol in an orbital shaker and agitated for two hours at two hundred revolutions per minute (Remi, Mumbai, India). After that, it was centrifuged at 4000 g for 10 min at 25 °C to separate the components. The residue was extracted again from the supernatant using the same method. Under decreased pressure at 45 °C, the supernatants were concentrated using a rotary evaporator (IKA Werke GmbH & Co., KG, Staufen, Germany) and freeze-dried using a Christ Beta 2–8 LD plus, Germany, as per the protocol [37].

### 2.4. Determination of Phenolic Compounds

Following the protocol, HPLC was used to separate the polyphenolic chemicals in the seed extract and identify the phenolic and flavonoid compounds [38]. A phenolic acid solution was fed into the machine to use HPLC., Shimadzu Class-VPV 5.03 (Tosoh Bioscience LLC, Kyoto, Japan), equipped with a UV-10 A Shimadzu detector, LC-16ADVP binary pump, DCou-14 A degasser, and C_18_ column (Sc 1011 No. H706081). The concentration of phenolic compounds was determined by measuring their retention duration and peak area.

### 2.5. Study Design

Thirty-six male “Sprague Dawley” rats weighed (150 ± 10 g) divided into six groups (6 rats per group). The first group was a control group, receiving only a baseline diet and distilled water. Alzheimer’s disease (AD) was induced in group 2 rats by oral administration (17 mg/kg body weight) of AlCl_3_ dissolved in distilled water [39] daily for four consecutive weeks. Rats in group 3 were supplemented concomitantly with both 500 mg/kg bw of an ethanolic extract of jambolan fruit orally once daily for 28 days [40] and oral supplementation of AlCl_3_ (17 mg/kg body weight). Rivastigmine (RIVA) aqueous infusion (0.3 mg/kg BW/day) was given orally to group 4 rats (as a reference drug) for 28 days [41] concomitantly with oral supplementation of AlCl_3_ (17 mg/kg body weight). Group 5 rats were orally treated with choline chloride (1.1 g/kg), as per Velazquez et al. [42], concomitantly with oral supplementation of AlCl_3_ (17 mg/kg body weight). Rats in Group 6 were given (500 mg/kg) of jambolan fruit ethanolic extract and (1.1 mg/kg) of choline orally for 28 days to test for additive effects concurrently with oral supplementation of AlCl_3_ (17 mg/kg bw).

### 2.6. Sacrifice and Sampling

The animals were fasted overnight and then exsanguinated to finish the experiment. Serum was separated from blood samples taken from individual rats by centrifugation at 3000 revolutions per minute (“r.p.m.”) for 10 min. To analyze the serum, it was separated, placed in sterile Eppendorf tubes, and frozen at −20 °C. Careful dissection was used to remove each rat’s brain before it was cleansed with saline solution (0.9% to eliminate sticky matter), dried with filter paper, and weighed. The brain was cut into quarters, with the first section frozen at −80 °C for RNA isolation and further molecular analysis, the second section fixed in 10% formalin for histopathological examination, the third section used fresh for the comet assay, and the fourth section homogenized for antioxidant analysis.

### 2.7. Growth-Related Parameters

Every week, feed intake (FI) was reported. The following equations were used to calculate growth-related parameters, such as final body weight gain (FBWG) and feed efficiency ratio (FER): Initial body weight (g)—final body weight (g) equals FBWG (g), and FER is equal to FBWG (g)/total FI (g).

### 2.8. Behavior Analysis: Testing for Mental Capacity through a Reinforcement System. The Morris Water Maze

The rats were taught to swim to a marked platform in a circular pool (180 cm in diameter and 60 cm in height) in the testing room. The platform provided the rats with a means of evading the water. The rats eventually figured out how to get to the platform from anywhere around the pool. The pool was sectioned off into four equal halves and filled to a depth of 40 cm. During the acquisition phase, a 9-cm-diameter circular, moveable platform was installed about 2 cm above the water level in one of the pool’s four corners. During the retention period, a comparable platform was submerged two centimeters below the pool’s water level. To make the water more difficult to see, a non-toxic color was added, and four spots were chosen randomly from the pool’s perimeter (North, South, East, and West) to serve as initial points for the acquisition process [43].

### 2.9. Preparation of Brain Homogenates and Biochemical Analysis

An ice-cold saline solution was used to clean the cerebral cortex and cerebellum. A 1:10 (*w*/*v*) dilution of ice-cold KCL buffer was used to homogenize brain tissue (1, 15 percent; pH 7.2). Post-mitochondrial supernatant (PMS) was obtained by centrifuging the homogenate at 10,000 g for 10 min at 4 °C to measure acetylcholine esterase (ACHE) as described previously [41]; according to Sasa and Blank (1977), dopamine (DA), serotonin (ST), and acetylcholine (ACH) [44], and acetylcholine (ACH) according to [45]. Total homocysteine (tHcy), interleukin-6 (IL-6), and tumor necrosis factor (TNF) serum levels were measured [46,47]. Superoxide dismutase (SOD) and other antioxidant markers in brain tissue were measured using a technique described by Nandi and Chatterjee (1988), while determining CAT activity according to ref. [48]. Malondialdehyde (MDA) and nitric oxide (NO) levels were assessed using the procedures given, whereas catalase (CAT) activity was measured based on refs. [49,50], respectively.

### 2.10. Histopathological Examination

Rat brain and cerebellar samples were obtained from each experimental group and preserved in 10% formalin. They were given a quick rinse with running water and then dehydrated by soaking in a bath of progressively more concentrated solutions of alcohol (methyl, ethyl, and absolute ethyl). After being cleaned with xylene, the samples were embedded in paraffin at 56 °C. Histopathological examination under a light microscope was performed by cutting sections (4 m thick), deparaffinizing them, and staining them with hematoxylin and eosin [51].

### 2.11. Gene Expressions Using Real Time-PCR

Total RNA was isolated from 100 mg of brain tissue using TRIzol (Invitrogen, Life Technologies, Carlsbad, CA, USA), and Nanodrop was used for quantification (Uv–Vis spectrophotometer Q5000/Quawell, San Jose, CA, USA). DNA was synthesized from RNA samples having an A260/A280 ratio of 1.8 or above using a cDNA synthesis kit (Fermentas, Waltham, MA, USA). SYBR Green master mix (Roche, Basel, Switzerland) and the primers listed in Table S1 were used for cDNA amplification. The conditions of quantitative RT-PCR amplification commenced with initial reverse transcription for the cDNA synthesis for 10 min at 55 °C. The resulting cDNA underwent 40 cycles of PCR with denaturation at 95 °C for 5 s, annealing at 55 to 58 °C for 25 s, and extension at °C for 15 s using the common housekeeping gene glyceraldehyde-3-phosphate dehydrogenase (GAPDH). Rotor-Gene Q (Qiagen, Valencia, CA, USA) was used to gather data and mechanically assess the threshold cycle value (Ct). 2−ΔΔ methods were applied to the analysis of amplification data [52].

### 2.12. Statistical Analysis

“Statistical Package for the Social Sciences (SPSS) for Windows (Version 20; Untitled—SPSS Data Editor) was used for all statistical analysis. The mean and standard deviation (mean SD) were used to summarize the data. One-way analysis of variance (ANOVA) and categorical data analysis were used to examine the data. The Duncan test was used to determine if the mean changes were statistically significant at the *p* < 0.05 level.

## 3. Results

### 3.1. The Polyphenolic Compounds of Bulb Extract

**Figure 1:**
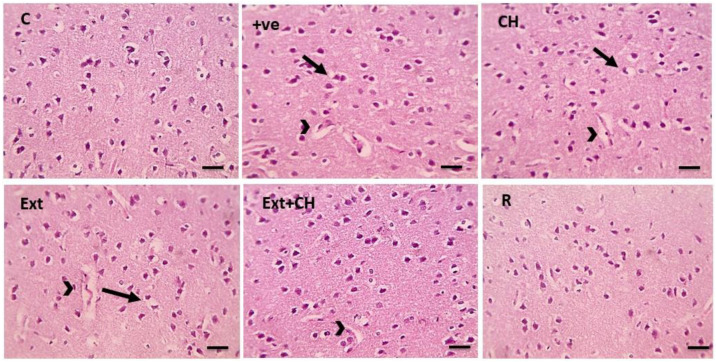
Microscopic pictures of HE-stained cerebral cortical sections x400 showing normal neurons in the control group (C). Cerebral cortical cells from the +ve group showed marked perineuronal (black arrows) and perivascular (arrowheads) edema. Cerebral cortical sections from the choline (CH) group showed moderate perineuronal (black arrows) and perivascular (arrowheads) edema. Cerebral cortical sections from fruit extract (EXT)group mild perineuronal (black arrows) and perivascular (arrowheads) edema. Cerebral cortical sections from the Fruit extract + choline (Ext + CH) group had milder perivascular edema (arrowheads). Cerebral cortical sections from Rivastgmine (R) group show an improved histological picture.

Data in Table 1 and Figure 1 revealed that the most abundant components of Jambolan bulb extract were gallic acid, catechin, p-hydroxybenzoic, and cinnamic (838.878, 73.115, 29.136, and 20.253 μg/g, respectively), followed by syringic, ferulic, vanillic, chrysin, and protocatechuic (15.381, 11.612, 8.321, 7.682, and 6.126 (μg/g), respectively (see Supplementary Figures S1 and S2).

### 3.2. Morris Water Maze Test

This study confirms previous research showing that male albino rats’ cognitive abilities diminish after receiving a chronic oral injection of AlCl_3_. Morris water maze performance was significantly worse (*p* < 0.05) in AlCl_3_-treated rats than in controls. AlCl_3_ was found to substantially impair learning and memory, although all groups given either jambolan extract, rivastigmine, choline, or a combination of the two were protected considerably. When tested against AlCl_3_-induced toxicity, rivastigmine and a mixture of choline and fruit extract fared better than either treatment alone (Table 2).

### 3.3. Body and Brain Weight

The AlCl_3_ group significantly (*p* < 0.05) underperformed the control group in terms of feed intake (FI), body weight growth (BWG percent), and feed efficiency ratio (FER). In contrast, the AlCl_3_-treated group saw a substantial (*p* < 0.05) increase in these parameters after receiving jambolan extract, rivastigmine, choline, and (extract + choline). The percentage of brain weight was not significantly different across all experimental groups (Table 3).

### 3.4. Effect of Jambolan Extract and Choline on Acetyl Choline (pg/mL), Dopamine (ng/mL) and Serotonin (pg/mL) in Rats Intoxicated by AlCl_3_

Serum biochemical characteristics for the investigated groups are listed in Table 4, and compared with the control group, acetylcholine (ACH), dopamine, and serotonin levels decreased after an oral dose of AlCl_3_. Serum levels of ACH, dopamine, and serotonin were suppressed by AlCl_3_ but were restored after oral administration of jambolan extract, rivastigmine, choline, and (extract + choline).

### 3.5. Effect of Jambolan Extract and Choline on Acetylcholine Esterase (mU/mL), IL6 (pg/mL), TNF (pg/mL), and HCY(pmol/mL) in Rats Intoxicated by AlCl_3_

ACE, IL6, TNF, and HCY levels illustrated a substantial (*p* < 0.05) increase in the AlCl_3_-treated group associated with the control group. However, the administration of jambolan extract, rivastigmine, choline, and (extract + choline) resulted in a considerable (*p* < 0.05) reduction of these parameters in the AlCl_3_-treated group (Table 5).

### 3.6. Effect of Jambolan Extract and Choline on MDA, NO, CAT, and SOD Levels in Alzheimer’s Disease Induced by AlCl_3_

We measured lipid peroxidation levels to investigate oxidative stress damage in brain tissue. Malondialdehyde (MDA) and nitric oxide (NO) levels were significantly elevated in the AlCl_3_-treated group (*p* < 0.05), which were significantly reduced by the administration of jambolan extract, rivastigmine, choline, and (extract + choline). Antioxidant enzymes such as superoxide dismutase (SOD) and catalase (CAT) are crucial in eliminating reactive oxygen species and protecting against oxidative stress. After AlCl_3_ exposure, enzyme levels for SOD and CAT dropped dramatically. The administration of jambolan extract, rivastigmine, choline, and (extract + choline) reversed these effects, as shown in Table 6.

### 3.7. Histopathological Results

**Figure 2:**
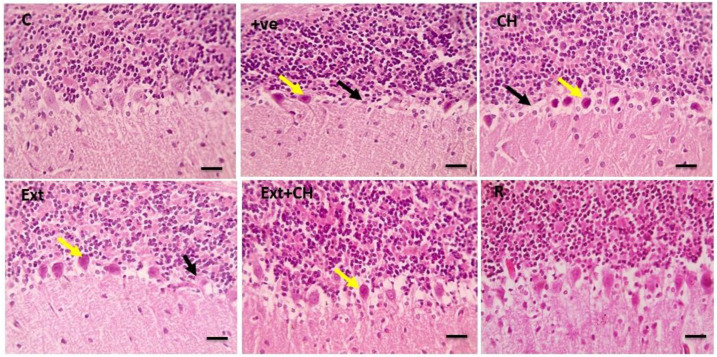
Microscopic pictures of HE-stained cerebellar sections x400 showing standard Purkinje layer in the control group (C). Cerebellar sections from the +ve group showed a significant loss of Purkinje fibers (black arrows) with degenerated neurons (yellow arrows). Cerebellar sections from the choline (CH) group showed marked focal loss of Purkinje fibers (black arrows) with many degenerated neurons (yellow arrows). Cerebellar sections from the fruit extract (EXT)group show considerable focal loss of Purkinje fibers (black arrows) with some degenerated Purkinje neurons (yellow arrows). Cerebellar sections from fruit extract + choline (Ext + CH) showing few degenerated Purkinje neurons (yellow arrows). Cerebellar sections from the rivastigmine (R) group show normal Purkinje neurons.

The control group showed normal neurons, while the positive control group showed marked perineural and perivascular edema; the choline-treated group and the fruit extract group showed mild perineuronal and perivascular edema, and this improved by co-administration of both fruit extract and choline as shown in Figure 2.

The cerebral tissue showed a standard Purkinje layer in the control group that was lost in the positive control group, and the choline-treated group showed significant focal loss of Purkinje fibers that was characterized by mild degeneration in the fruit extract-treated group with substantial improvement in the co-administration of both fruit extract and choline.

**Figure 3:**
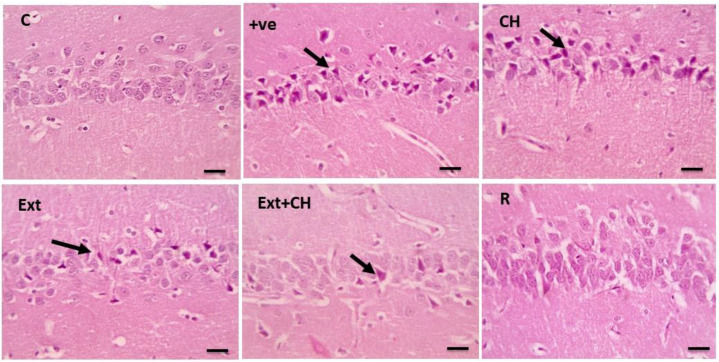
Microscopic pictures of HE-stained hippocampal sections x400 showing normal neurons of the pyramidal layer in the control group (C). Cerebral cortical sections from the (+ve) group show many shrunken and degenerated neurons (black arrows). Cerebral cortical sections from choline (CH) show many shrunken and degenerated neurons (black arrows). Cerebral cortical sections from the fruit extract (EXT)group showed few shrunken and degenerated neurons (black arrows). Cerebral cortical sections from the Fruit extract + choline (Ext + CH) group have few shrunken and degenerated neurons. Cerebral cortical sections from the rivastgmine (R) group show an improved histological picture.

Figure 3 showed that the hippocampal sections had a standard pyramidal layer in the control group, which was shrunken and degenerated in the control-positive one, while the choline-treated one showed mild degenerative changes of neurons as well; the fruit extract groups showed significant improvement in the co-administration of both fruit extract and choline.

### 3.8. Effect of Jambolan Extract and/or Choline Treatment on Inflammatory mRNA Gene Expression in AlCl_3_ Treated Rats

**Figure 4:**
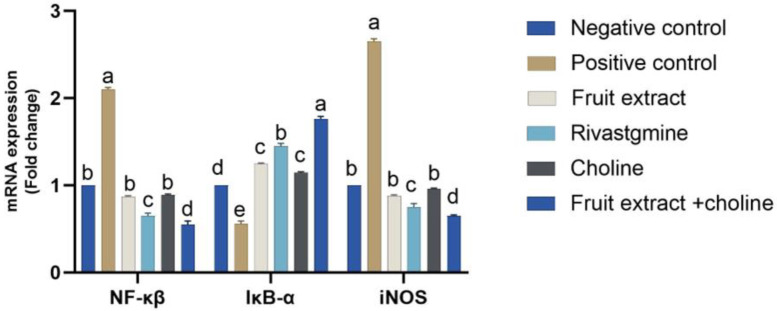
Effects of Jambolan extract and/or choline on the mRNA transcription of inflammatory-related signaling pathways (iNOS, NF-κβ, and IκB-α). Data were presented as mean ± SD. Different superscripts ^a–d^ indicated significant differences within the column. A significant *p*-value < 0.05.

Figure 4 shows the effect of the administration of Jambolan extract and/or choline on the mRNA expression of inflammatory-related signaling pathways (iNOS, NF-κβ, and IκB-α) in control and treatment groups. There was an AlCl_3_-treated decrease in IκB-α mRNA expression (*p* < 0.05) AlCl_3_ treated rats, accompanied by significant upregulation of both iNOS and NF-κβ in the other treated groups. Conversely, treatment with Jambolan Extract and/or choline significantly reversed the AlCl_3_ effect, with noticeable improvement in the co-treatment of Jambolan and choline concerning other treated groups.

## 4. Discussion

There is currently no established underlying mechanism for Alzheimer’s disease (AD), a primary severe neurodegenerative condition of the brain. One of the significant obstacles in neurology is finding an effective treatment for it. Therefore, it is still linked to a relatively high death and morbidity rate. Neurofibrillary tangles (NFTs) and senile plaques are two characteristic brain lesions of Alzheimer’s disease. Amyloid-beta peptide (A) is present in senile plaques, and NFTs are generated when abnormally hyperphosphorylated tau protein accumulates intracellularly to form paired helical filaments [53].

Gallic acid, catechins, cinnamic acid, syringic acid, ferulic acid, and vanillic acid were all identified in high concentrations in the jambolan bulb extract, as had been predicted by the study. This finding accords with a prior study by Ahmed et al. [54], which found that *S. cumini*, the plant the extract comes from, has a high polyphenol content. In addition to ellagic acid, triterpenoids, oleanolic acetyl acid, quercetin, isoquercitrin, myricetin, and kaempferol, the fruit and flowers of *S. cumini* are rich in anthocyanins such as cyanidin, delphinidin, peonidin, pelargonidin, petunidin, and malvidin. In related research by Rajan et al. [55], according to high-performance liquid chromatography (HPLC), the chlorogenic acid, catechin, and epicatechin content of the ethanolic extract of *S. cumini* is higher at 80% than in the jambolan extract at 20%. Furthermore, Brusamarello et al. [56] found that jambolan’s antioxidant activity is a result of the presence of bio-polyphenols such as gallic acid, quinic acid, protocatechuic acid, glycosylated anthocyanins, myricetin isomers, and proanthocyanidins like epigallocatechin trimers.

Literature reports referenced in the present study corroborated the findings that chronic oral treatment of AlCl_3_ leads to the degradation of learning and memory functions in male albino rats. In the Morris water maze test, AlCl_3_-treated animals performed significantly worse than controls (*p* < 0.05) due to impairments in learning and memory. All treated groups, including the AlCl_3_ treated rats, exhibit substantial resistance to the learning and memory impairments caused by AlCl_3_. Consistent with these findings, Naghizadeh and Mansouri’s [57] jambolan extract’s gallic acid has been credited with its antioxidant and neuroprotective effects, which protect against neurotoxicity and excitotoxicity after brain injury. Prema et al. [58] suggested that AlCl_3_-treated mice completed the Morris water maze test faster, a more sensitive measure of hippocampus function. On day 25, the AlCl_3_ group took significantly longer than the control group to reach the visible platform in the Morris water maze test, demonstrating memory impairments and impaired spatial memory. Insight into the precise mechanism is provided by Mohapatra et al. [59]; they found that rats exposed to AlCl_3_ spent considerably more time running onto the Morris water maze platform, demonstrating an impairment in spatial memory. These symptoms have previously been linked to cognitive and memory impairments. Our finding was in line with Ogunlade et al. [35], who reported the protective effect of gallic acid against the neurodegeneration induced by aluminum chloride. In addition, choline is a vital ingredient for all living things, and it can be ingested or produced through phosphatidylcholine breakdown [60]. Maintaining proper brain function also requires an adequate quantity of choline [61]; this explains our result regarding the Jambolan extract and choline-treated rats, which showed a significantly increased body weight of the other treated groups.

Consistent with prior literature studies, the study indicated that male albino rats given AlCl_3_ orally over time had a deterioration in their learning and memory capacities. Compared with the control group, the rats given AlCl_3_ alone showed significant impairments in learning and memory on the Morris water maze test (*p* < 0.05). AlCl_3_-induced deficits in learning and memory were prevented in all treated groups. Naghizadeh and Mansouri [57] have previously reported that the antioxidant and neuroprotective properties of jambolan extract, mainly due to gallic acid, may prevent neurotoxicity and excitotoxicity resulting from brain injury. Acetylcholine, a neurotransmitter crucial for learning, memory, muscular control, and mood, is synthesized in the body from choline, making it an essential vitamin [62]. Acetylcholine stimulates microglial alpha7 nicotinic acetylcholine receptors (7nAchR) [63]. However, with AD, there is an increase in activated microglia, leading to chronic brain inflammation, which ultimately destroys neurons [64]. Increasing acetylcholine may thereby activate alpha 7 nAChR, leading to a reduction in the number of activated microglia. An additional amount of choline in the diet is a potential therapy for AD [42]; this explains our result in which Jambolan extract and choline-treated rats showed marked increases in acetylcholine, serotonin, and dopamine in the other treated groups that either received Jambolan extract or choline. This explains the promoting effect of both Jambolan extract and choline and improved spatial memory. In addition, catechin is one of the Jambolan extracts found to have an enhancing effect on brain function through modulation of cholinergic neurotransmission [65]. In addition, catechin has shown promise as an adjuvant therapy for the treatment of DOX-induced cognitive impairment, and this could lead to an improvement in survivors’ quality of life. Possible explanations for this enhancement include enhanced antioxidant defense, suppression of neuroinflammation, and acetylcholine esterase enzyme inhibition [66]. In the same line, *p*-hydroxybenzoic, cinnamic acid, and chrysin possess a concentration-dependent pattern of powerful inhibitory effects on brain acetylcholinesterase [67].

Relatedly, Prema et al. [58] showed that rats given AlCl_3_ performed better on a test of hippocampus function called the Morris water maze, indicating that the chemical had a beneficial effect on the animals’ ability to navigate the maze. Morris water maze times for AlCl_3_-treated rats were significantly longer than controls beginning on day 25, demonstrating memory and spatial memory impairment [59]. Moreover, the Morris water maze test revealed that AlCl_3_ considerably increased the escape delay, indicating a loss in spatial memory. Problems in thinking and remembering have been connected to these findings. The current results align with those in Ogunlade et al. [35], who found that gallic acid-rich jambolan extract significantly mitigated these deficiencies when supplied alongside AlCl_3_.

Intoxicated rats from the current study had less body weight gain throughout the trial when given aluminum chloride. The findings are consistent with Buraimoh [68]. Weight loss is defined as the decrease in total body mass due to a mean loss of fluid, body fat, or adipose tissue and lean mass, which includes bone mineral deposits, muscle, tendon, and other connective tissue, for the sake of medicine, health, or physical fitness. It can happen accidentally due to sickness or on purpose to combat actual or perceived obesity. Many medical disorders and diseases, even life-threatening ones like cancer and AIDS, can cause a person to lose weight unexpectedly. Diabetes, some drugs, dehydration, and other causes can all lead to fluid loss and weight loss caused by decreased fat and lean mass. Cachexia risk increases with dehydration, fat loss, and muscle waste. These results are consistent with Balgoon [69]; they found that rats’ weight gain was significantly (*p* < 0.001) reduced when given AlCl_3_ over eight weeks compared with rats given a placebo. Yet another investigation by Lahouel et al. [70] demonstrated striking long-term effects on body and brain weight following a weekly intraperitoneal injection of aluminum; these results indicated that AlCl_3_ interaction with hormone status or protein synthesis was responsible for the observed changes. Aluminum compounds impede stomach emptying and reduce gastrointestinal motility in humans and rats, which may explain why taking them causes weight loss. However, rats given aluminum chloride intoxication lost weight throughout the study, which scientists attribute to the metal’s anorectic effect because it inhibits the production of serotonin and dopamine. These two neurotransmitters are crucial in regulating eating habits and the sensation of fullness [71].

Consistent with prior literature reports, this study demonstrated that giving aluminum chloride to rats caused them to lose weight [68], highlighting that losing weight can result from a medical condition or a deliberate attempt to remedy one’s overweight or obese state. The results of the current study agree with those of Han et al. [72]; it showed considerable weight loss and aluminum’s impairment of normal metabolism after chronic exposure in rats exposed to AlCl_3_. Protein and fat oxidation facilitate weight loss by blocking glycolysis and the Krebs cycle [73].

Furthermore, the study indicated that compared with the negative control group, the positive control group’s acetylcholine levels were lower and their acetylcholinesterase (AChE) levels were higher after the oral administration of aluminum chloride. The results are consistent with Kaushik et al. [74], who found that the loss of cholinergic neurotransmitters due to the constant activity of AChE is a significant cause of cognitive impairments in Alzheimer’s disease. In Nordberg et al. [75], it was also shown that butyrylcholinesterase (BuChE) hydrolyzes acetylcholine in cases with low levels of AChE, revealing the centrality of acetylcholine to the cholinergic system and memory processes. AlCl_3_ has been observed to reduce cholinergic neurotransmission and enhance acetylcholinesterase activity in animal studies [76]. In addition, acetylcholine synthesis is boosted by choline [77]. The previous literature proved the promoting effect of the co-treatment of Jambolan extract and choline-treated.

According to the results of this investigation, elevated levels of AChE activity may have a role in the development of Alzheimer’s disease (AD) [78]. Restoring short- and long-term memory in patients with Alzheimer’s disease using therapeutic approaches that target AChE and BuChE has been observed [74]. According to rivastigmine is a cholinesterase inhibitor that increases synaptic acetylcholine activity [79]. AlCl_3_ was discovered to increase AChE and BuChE movements in the brains of albino rats. The cholinesterase activities, however, were kept at levels like the negative control when AlCl_3_ was co-administered with jambolan extract. Jambolan extract contains gallic acid, an enzyme inhibitor that increases acetylcholine availability at synapses and, by extension, improves cognitive function in experimental animals. Gallic acid’s anticholinesterase actions in this investigation are consistent with previous research [80].

Deficits in various neurotransmitters, in addition to acetylcholine, have been linked to the neurodegenerative symptoms of Alzheimer’s disease, as previously discussed [81]. According to reports, norepinephrine plays a role in the brain’s memory, learning, and attention systems [82]. Alzheimer’s disease is linked to low levels of dopamine and acetylcholine, as well as memory loss, according to research conducted in animal models [83].

Aluminum chloride treatment significantly decreased serotonin and dopamine levels, while other treatment groups boosted these neurotransmitters. The results align with those of Butzlaff and Ponimaskin [84], who noted a negative correlation between norepinephrine concentrations and mental acuity. A decrease in brain serotonin levels has been found in Alzheimer’s patients, even though serotonin is the most abundant neurotransmitter in the central nervous system and plays a critical role in learning and memory function [85].

Pan et al. [86] elucidated the connection between amyloid aggregation and dopamine impairment. Dopamine is implicated in the etiology of Alzheimer’s disease since it has been shown to improve cognitive function in rats given a dopamine supplement. According to this interpretation, Ogunlade et al. [35] found that norepinephrine, serotonin, and dopamine levels were all significantly lowered by AlCl_3_. Jambolan extract, rich in polyphenols such as gallic acid, increases levels of these neurotransmitters when taken together with AlCl_3_. In addition, when gallic acid was given to rats, both norepinephrine and dopamine levels increased noticeably. These findings are consistent with previous research showing that gallic acid’s neuroprotective effects involve their ability to modulate levels of various neurotransmitters, suggesting that gallic acid may alleviate neurodegenerative symptoms in Alzheimer’s disease caused by disturbances in neurotransmitter levels.

Neurodegeneration on par with Alzheimer’s disease may result from aluminum’s generation of free radicals in the brain. This is especially true due to the brain’s increased ROS level and rapid oxygen consumption [87], causing a decline in antioxidant activity, which has been linked to toxicity and the onset of neurodegeneration [88]. Therefore, diseases in which neurotoxicity is suspected may benefit from antioxidant therapy against oxidative stress.

The levels of MDA and NO were observed to be significantly elevated in the AlCl_3_ group but were dramatically reduced by the extract, rivastigmine, choline, and extract + choline. Protecting cells from oxidative stress and removing reactive oxygen species requires antioxidant enzymes like superoxide dismutase (SOD) and catalase (CAT). The enzymes SOD and CAT were dramatically inhibited after being exposed to AlCl_3_. Tsaluchidu et al. [89] found decreased catalase and SOD activity in the brain as evidence of oxidative stress caused by AlCl_3_ exposure. Increased lipid peroxidation is a crucial mechanism by which degenerative change is expressed in the Alzheimer’s disease brain, and both high lipid content and double bonds in brain membrane phospholipids contribute to this process. Moreover, previous research found that aluminum-induced lipid peroxidation significantly increased brain MDA levels, as reported by Nehru and Anand [90]. Abdel-Salam et al. [91] confirmed, in line with prior research, that AlCl_3_ treatment increased brain NO levels. Likewise, Oyetayo et al. [92] showed that thee oxidation of stored H_2_O_2_ in the brain by AlCl_3_ decreased total thiol levels. High amounts of MDA and low levels of total thiol were found in the brains of AlCl_3_-exposed rats. The antioxidant state of the rat brains was dramatically enhanced after treatment with jambolan extract, which contains gallic acid. The much lower levels of malondialdehyde and nitric oxide, the significantly higher activities of superoxide dismutase and catalase, and the significantly higher levels of total thiol all suggested that gallic acid exerted a significantly greater antioxidant effect. Previous research has shown that NO has neuroprotective properties in the central nervous system, and it has also been linked to memory and learning processes [93].

Aluminum poisoning disrupts all three nitric oxide-producing isoforms, increasing NO, which has been linked to neurodegeneration via several pathways (such as oxidative and nitrative stress). According to this theory, peroxynitrite anion (ONOO) is produced when nitric oxide (NO) reacts with superoxide anion radicals at high concentrations [87]. However, when AlCl_3_ and jambolan extract were given together, brain nitric oxide levels were dramatically lowered compared with when only AlCl_3_ was provided together. Both therapies in this study significantly reduced nitric oxide levels, suggesting they may mitigate the nitrative stress caused by peroxynitrite in the brain. The findings of this study support previous claims that combination medication may be more effective than monotherapy [94,95,96,97]. Treatment with jambolan extract containing gallic acid considerably improved the brain’s antioxidant status compared with treatment with aluminum chloride alone in the animals utilized in this investigation. Additionally, gallic acid’s (a polyphenolic component) natural antioxidant potential may contribute to its impressive capability to reduce oxidative stress [34].

Due to its high oxygen consumption and low antioxidant defenses, the brain is particularly vulnerable to oxidative stress. Thus, significant neuronal damage due to AlCl_3_ neurotoxicity results from an imbalance in the antioxidant defense system. Symptomatic of the onset of Alzheimer’s disease and linked to cognitive decline, these conditions are documented by Parekh et al. [98]. Malondialdehyde disrupts neuronal signaling and hippocampus structure by forming harmful protein adducts through covalent protein binding [99]. Decreased levels and activity of antioxidants like SOD and CAT have also been linked to Alzheimer’s disease, according to several studies [100,101]. In addition, Nabila [102] and Shi [103] reported choline’s antioxidant capacity, strengthening the positive effect of the co-treatment of jambolan extract and choline.

To protect neurons from oxygen-free radical damage, catechin and p-hydroxybenzoic are potent antioxidants that boost SOD activity in brain astrocytes [104,105]. As well as cinnamic acid decreased serum levels of CK-MB, LDH, TNF-α, and IL-6, and myocardial ischemia increased serum NO activity. As well as increased SOD activity and decreased MDA content in the heart [106].

Treatment with jambolan extract reversed the AlCl_3_-induced dysregulation of antioxidant systems in the brains of albino rats. These results support the theory that boosting the brain’s antioxidant systems could effectively deal with neurotoxic diseases like Alzheimer’s [87]. This aligns with previous research showing that conventional medicines and phenolic substances reduce oxidative stress in the brains of Alzheimer’s disease rats [107,108]. Gallic acid, a significant component of jambolan extract, has been hypothesized to have an antioxidant effect because of its potential to activate Nrf2, a critical regulator of the antioxidant system [109]. Memory and learning problems may originate in the inflammation early in Alzheimer’s [110].

The aqueous jambolan seed extract contains several phytochemicals that may have antigenotoxic effects, including gallic acid, ferulic acid, caffeic acid, rutin, and quercetin. To prevent carcinogen-induced DNA damage, they strengthen the body’s antioxidant defenses. Dried jambolan seed extract (prepared with 60% methanol as a solvent) suppressed the expansion of VEGF-induced breast cancer cells [111]. Regarding the same dose, Yassin and El-Moslemany [40] observed that the natural antioxidants present in a methanolic extract of jambolan fruits (500 mg/kg body) can prevent kidney and testicular tissue from being damaged by CCl_4_-induced oxidative stress. Due to its intense anthocyanin concentration, jambolan fruit is rich in antioxidants and free radical scavengers.

The current study found that, compared with the control group, the AlCl_3_-treated group had considerably higher levels of IL6, TNF-α, and HCY. In contrast, the AlCl_3_-treated group saw a substantial decrease in these measures after receiving jambolan extract treatment. Previous studies indicated that the inflammatory cytokines IL-6, IL-1, and TNF-α are implicated in mechanisms leading to the phosphorylation of tau proteins and neuronal death. Therefore, these results make sense [112].

Both increased IL-6 synthesis and elevated IL-1 levels were observed to block cholinergic systems [113]. Consistent with these results, co-administration of AlCl_3_ and jambolan extract (which includes the primary polyphenol gallic acid) reduced the increase in IL-6, IL-1β, and TNF- levels in the brains of albino rats [114]. These findings highlight gallic acid’s anti-inflammatory capacity, which is crucial because anti-inflammatory medications have been shown to reduce the risk of developing Alzheimer’s disease by up to 50%. The release of more pro-inflammatory cytokines exacerbates the inflammatory response, and metals such as aluminium have been proven to boost TNF-, which encourages the recruitment of leukocytes [115]. Furthermore, p-Coumaric acid (p-CA), a polyphenol, has decreased inflammatory molecular alterations like TNF-α, suggesting it may be helpful as an alternate treatment for refractory depression [116]. Choline supplementation protects the brain from Alzheimer’s disease by decreasing the microglia activation. 

A decrease in the amyloidogenic processing of APP was observed in female APP/PS1 mice when they were given choline supplements throughout their lives. Increasing maternal choline consumption has significantly decreased the A load in APP/PS1 mice [117]. By affecting homocysteine (Hcy), which binds to Aβ and facilitates its aggregation and accumulation, it may be possible to reduce the A burden mechanistically—Choline’s metabolite, betaine, transfers methyl groups from Hcy to methionine [118]. Thus, decreased Hcy levels may contribute to less Aβ aggregation and accumulation in APP/PS1 mice exposed to lifelong choline supplementation [42].

The histopathological analysis revealed that the negative control group’s cerebral cortex, cerebellar region, and hippocampus displayed normal neurons compared with the aluminum chloride-treated groups. This result was in line with Liaquat et al. [119]. In the AlCl_3_-intoxicated rats, the cerebral cortex neurons were loosely packed, irregular in shape, and darkly stained. Correspondingly, Al-Hazmi et al. [120] showed that the cerebellar cortex cells were disorganized and had eccentric nuclei, rough formation, localized gliosis, and cerebral vascular congestion due to AlCl_3_ administration.

Memory storage and recall, context representation, and navigation are just a few of the cognitive tasks in which the hippocampus plays a pivotal role. The hippocampus’s main pathways for processing information are the dentate gyrus’s granule cells and the cornu ammoni’s pyramidal cells [121]. Deficits in memory and learning processes related to the hippocampus have been linked to aluminum accumulation in the prefrontal cortex and hippocampus [110]. Increased apoptosis in the hippocampus and cortex via up-regulation of proapoptotic proteins is a process in which oxidative stress is implicated, and chronic treatment of AlCl_3_ in rats has been shown to induce decreased pyramidal cells and altered morphology of disordered hippocampal cells [122].

These results emphasize the need to shield the hippocampus from aluminum’s destructive effects. Rats given AlCl_3_ showed improved hippocampal cell population and arrangement after treatment with jambolan extract and gallic acid, suggesting that these substances may prevent or reduce the adverse effects of aluminum buildup on the hippocampus [87]. To prevent and treat cognitive deficits associated with the hippocampus, it is crucial to learn more about the mechanisms underlying the protective effects of these chemicals on the hippocampus.

In the current work, co-administration of AlCl_3_ with jambolan extract and gallic acid increased hippocampus cell population and organization in rats. Perhaps gallic acid’s capacity to reduce oxidative stress in the brain and stop apoptosis in the hippocampus due to oxidative stress plays a role in this [87]. When comparing the AlCl_3_ group, which showed many normal neurons accompanied by glial cells, to the control group, which showed cell degeneration, vacuolation, necrosis, and fibrosis in the cerebral cortex, it is clear that the AlCl_3_ group experienced cell death [71]. Histological investigations show that exposure to AlCl_3_ for eight weeks causes significant neurodegeneration in the hippocampus, similar to the present study’s results [123]. Furthermore, the present study’s histological analyses revealed that AlCl_3_ decreased the number of viable cells and had a sparse distribution. The prefrontal cortex of rats given jambolan extract, gallic acid, and AlCl_3_ demonstrated increased cell viability and equal cell distribution. Protection against caspase-3-mediated apoptosis in AlCl_3_-induced Alzheimer’s disease in rats has been shown by gallic acid [124]. The transcription factor NF-κB plays a vital role in the inflammatory process following Alzheimer’s through upregulation of the proinflammatory cytokines [125]. AlCl_3_-persuaded inhibitor κB-α (IκB-α) degradation and subsequently activation of NF-κB, which was reversed by the Jambolan Extract and/or choline, with a marked improvement in the co-treatment of Jambolan and choline in other treated groups, our result was in line with Kumar et al. [126], as they proved the anti-inflammatory effect of the Jambolan Extract due to the antioxidants and immunomodulatory properties of its constituents. In the same way, Liu et al. [127] demonstrated the anti-inflammatory effect of choline through the inhibition of the inflammatory cytokines [128].

## 5. Conclusions

The present study revealed that AlCl_3_ has terrible neurotoxic consequences in the pathogenesis of Alzheimer’s disease, including degradation of learning and memory functions and oxidative stress. However, Jambolan bulb extract’s antioxidant and anti-inflammatory responses due to its significant components such as gallic acid, catechin, p-hydroxybenzoic, cinnamic, syringic, ferulic, vanillic, chrysin, and protocatechuic may have potential therapeutic applications in the prevention and treatment of hippocampus-related cognitive impairments and Alzheimer’s disease induced by AlCl_3_ through improving the neurotransmitters, decreasing the inflammatory cytokines IL6 and TNF-α, as well as modulation of inflammatory mRNA gene expression, in addition to improving the antioxidant capacity of CAT and SOD in ameliorating memory impairments and protecting neuronal cells from oxidative damage, mopping-up free radicals that cause neurodegenerative disease, and ultimately protecting the brain from the effects of Alzheimer’s disease. We conclude that treatment with jambolan extract alone or with choline may help prevent or mitigate the harmful effects of aluminum accumulation on the brain, especially in people most susceptible to AlCl_3_.

## Figures and Tables

**Table 1:** Phenolic compounds for Jampolan bulb extract (μg/g).

Compound	Concentration (μg/g)
Gallic	838.878
Protocatechuic	6.126
p-hydroxybenzoic	29.136
Gentisic	ND
Cateachin	73.115
Chlorogenic	ND
Caffeic	ND
Syringic	15.381
Vanillic	8.321
Ferulic	11.612
Sinapic	ND
Rutin	ND
p-coumaric	ND
Apigenin-7-glucoside	ND
Rosmarinic	ND
Cinnamic	20.253
Qurecetin	ND
Apigenin	ND
Kaempferol	ND
Chrysin	7.682

**Table 2:** Impact of Jambolan extract, choline, and the combination on the Morris water maze test for memory retention in rats.

Groups	Time (min)
Negative control	0.032 ± 0.004 ^c^
Positive control	0.94 ± 0.09 ^a^
Fruit extract	0.27 ± 0.04 ^b^
Rivastgmine	0.028 ± 0.04 ^c^
Choline	0.32 ± 0.08 ^b^
Fruit extract + choline	0.13 ± 0.03 ^c^

Data were shown as mean ± SD. Different superscripts ^a–c^ indicated significant differences within the same column. A significant *p*-value < 0.05.

**Table 3:** Impact of Jambolan extract and choline on FI (g/d), BWG%, FER, and brain weight % levels in rats intoxicated by AlCl_3_.

	BWG (%)	FI (g)/d	FER	Brain Weight%
Negative control	53.27 ± 1.98 ^a^	202.67 ± 6.81 ^a^	0.44 ± 0.02 ^a^	0.75 ± 0.01 ^b^
Positive control	18.97 ± 1.31 ^d^	94.97 ± 6.11 ^d^	0.25 ± 0.01^d^	0.76 ± 0.02 ^b^
Fruit extract	32.37 ± 9.93 ^c^	164.33 ± 4.04 ^b,c^	0.37 ± 0.01 ^b^	0.76 ± 0.02 ^b^
Rivastgmine	43.57 ± 0.74 ^b^	170.67 ± 4.62 ^b^	0.39 ± 0.01^b^	0.81 ± 0.02 ^a^
Choline	31 ± 1 ^c^	160 ± 8 ^c^	0.30 ± 0.01 ^c^	0.74 ± 0.01 ^b^
Fruit extract + choline	50.57 ± 1.25 ^a,b^	199.67 ± 1.53^a^	0.43 ± 0.02 ^a^	0.76 ± 0.01 ^b^

Data were presented as mean ± SD. Different superscripts ^a–d^ indicated significant differences within the same column. The significant *p*-value < 0.05.

**Table 4:** Effect of Jambolan extract and choline on Acetylcholine (pg/mL), Dopamine (ng/mL), and serotonin (pg/mL) in rats intoxicated by AlCl_3_.

	Acetylcholine (pg/mL)	Serotonin (pg/mL)	Dopamine (ng/mL)
Negative control	37.18 ± 4.82 ^a^	51.9 ± 3.08 ^a^	2.34 ± 0.11 ^a^
Positive control	9.8 ± 1.02 ^e^	17.83 ± 2.38 ^d^	0.81 ± 0.09 ^e^
Fruit extract	20.9 ± 2.14 ^c^	31.5 ± 3.10 ^c^	1.39 ± 0.06 ^c^
Rivastgmine	27.68 ± 1.86 ^b^	41.97 ± 2.78 ^b^	1.87 ± 0.09 ^b^
Choline	14.78 ± 2.39 ^d^	22.5 ± 2.81 ^d^	1.11 ± 0.12 ^d^
Fruit extract + choline	26.03 ± 4.75 ^b^	39.97 ± 4.64 ^b^	1.81 ± 0.21 ^b^

Data were presented as mean ± SD. Different superscripts ^a–e^ indicated significant differences within the same column. A significant *p*-value < 0.05.

**Table 5:** Effect of Jambolan extract and choline on Acetylcholine esterase (mU/mL), IL6 (pg/mL), TNF (pg/mL), and HCY (pmol/mL) in rats intoxicated by AlCl_3_.

	IL6 (Pg/mL)	TNF (Pg/mL)	HCY (pmol/mL)	Acetylcholine Esterase (mU/mL)
Negative control	1.18 ± 0.05 ^e^	32.43 ± 3.55 ^e^	73.73 ± 8.49 ^d^	11.48 ± 2.29 ^d^
Positive control	2.49 ± 0.13 ^a^	118.07 ± 6.41 ^a^	218.37 ± 10.69 ^a^	60.27 ± 6.03 ^a^
Fruit extract	1.69 ± 0.09 ^c^	70.07 ± 10.14 ^c^	170.63 ± 7.02 ^b^	30.13 ± 3.39 ^c^
Rivastgmine	1.44 ± 0.09 ^d^	54.53 ± 5.42 ^d^	118.58 ± 9.94 ^c^	15.58 ± 1.57 ^d^
Choline	1.89 ± 0.11 ^b^	95.6 ± 6.69 ^b^	183.88 ± 7.66 ^b^	42.5 ± 5.27 ^b^
Fruit extract + choline	1.46 ± 0.13 ^d^	56.23 ± 9.59 ^d^	120.58 ± 18.62 ^c^	16.03 ± 3.09 ^d^

Data were presented as mean ± SD. Different superscripts ^a–e^ indicated significant differences within the same column. A significant *p*-value < 0.05.

**Table 6:** Impact of Jambolan extract and choline on MDA, NO, CAT, and SOD levels in Alzheimer’s disease induced by AlCl_3_.

Groups	MDA (nmol/g)	NO (µmol/g)	CAT (U/g)	SOD (U/g)
Negative control	14.4 ± 1.96 ^d^	1.29 ± 0.14 ^e^	2.68 ± 0.12 ^a^	214.8 ± 8.09 ^a^
Positive control	55.9 ± 5.49 ^a^	4.15 ± 0.20 ^a^	0.94 ± 0.09 ^d^	75.88 ± 4.33 ^e^
Fruit extract	27.38 ± 3.89 ^c^	3.03 ± 0.31 ^c^	1.57 ± 0.25 ^c^	117.7 ± 4.05 ^c^
Rivastgmine	20.63 ± 3.79 ^c,d^	2.23 ± 0.55 ^d^	2.15 ± 0.30 ^b^	156.95 ± 8.80 ^b^
Choline	36.78 ± 6.72 ^b^	3.54 ± 0.22 ^b^	1.30 ± 0.09 ^c^	91.1 ± 3.44 ^d^
Fruit extract + choline	22 ± 3.55 ^c^	2.35 ± 0.42 ^d^	2.19 ± 0.13 ^b^	162.38 ± 7.49 ^b^

Data were presented as mean ± SD. Different superscripts ^a–e^ indicated significant differences within the same column. A significant *p*-value < 0.05.

## Data Availability

Available from the corresponding author upon reasonable request.

## Supplementary Materials

The following supporting information can be downloaded at: https://www.mdpi.com/article/10.3390/toxics11060509/s1, Figures S1 and S2: The Polyphenolic Compounds of Bulb Extract; Table S1: Primers for gene expression by RT-PCR

## References

[1] Naz F., Siddique Y.H. (2020). Human Brain Disorders: A Review. Open Biol. J..

[2] Lancaster E. (2016). The diagnosis and treatment of autoimmune encephalitis. J. Clin. Neurol..

[3] Tai J., Liu W., Li Y., Li L., Hölscher C. (2018). Neuroprotective effects of a triple GLP-1/GIP/glucagon receptor agonist in the APP/PS1 transgenic mouse model of Alzheimer’s disease. Brain Res..

[4] Uwishema O., Mahmoud A., Sun J., Correia I.F.S., Bejjani N., Alwan M., Nicholas A., Oluyemisi A., Dost B. (2022). Is Alzheimer’s disease an infectious neurological disease? A review of the literature. Brain Behav..

[5] Edwards G.A., Gamez N., Escobedo G., Calderon O., Moreno-Gonzalez I. (2019). Modifiable risk factors for Alzheimer’s disease. Front. Aging Neurosci..

[6] Moreno-Gonzalez I., Morales R., Baglietto-Vargas D., Sanchez-Varo R. (2020). Risk Factors for Alzheimer’s Disease. Front. Aging Neurosci..

[7] Amtul Z., Yang J., Lee T.-Y., Cechetto D.F. (2019). Pathological changes in microvascular morphology, density, size and responses following comorbid cerebral injury. Front. Aging Neurosci..

[8] Livingston G., Huntley J., Sommerlad A., Ames D., Ballard C., Banerjee S., Brayne C., Burns A., Cohen-Mansfield J., Cooper C. (2020). Dementia prevention, intervention, and care: 2020 report of the Lancet Commission. Lancet.

[9] Ali A.A., Ahmed H.I., Khaleel S.A., Abu-Elfotuh K. (2019). Vinpocetine mitigates aluminum-induced cognitive impairment in socially isolated rats. Physiol. Behav..

[10] Borai I.H., Ezz M.K., Rizk M.Z., Aly H.F., El-Sherbiny M., Matloub A.A., Fouad G.I. (2017). Therapeutic impact of grape leaves polyphenols on certain biochemical and neurological markers in AlCl3-induced Alzheimer’s disease. Biomed. Pharmacother..

[11] Castelli V., Benedetti E., Antonosante A., Catanesi M., Pitari G., Ippoliti R., Cimini A., d’Angelo M. (2019). Neuronal cells rearrangement during aging and neurodegenerative disease: Metabolism, oxidative stress and organelles dynamic. Front. Mol. Neurosci..

[12] Pereira A.C., Gray J.D., Kogan J.F., Davidson R.L., Rubin T.G., Okamoto M., Morrison J.H., McEwen B.S. (2017). Age and Alzheimer’s disease gene expression profiles reversed by the glutamate modulator riluzole. Mol. Psychiatry.

[13] Raschetti R., Albanese E., Vanacore N., Maggini M. (2007). Cholinesterase inhibitors in mild cognitive impairment: A systematic review of randomised trials. PLoS Med..

[14] Beheshti S., Aghaie R. (2016). Therapeutic effect of frankincense in a rat model of Alzheimer’s disease. Avicenna J. Phytomed..

[15] Ravi S.K., Ramesh B.N., Mundugaru R., Vincent B. (2018). Multiple pharmacological activities of Caesalpinia crista against aluminium-induced neurodegeneration in rats: Relevance for Alzheimer’s disease. Environ. Toxicol. Pharmacol..

[16] Naber M., Hommel B., Colzato L.S. (2015). Improved human visuomotor performance and pupil constriction after choline supplementation in a placebo-controlled double-blind study. Sci. Rep..

[17] Melo E.d.A., Maciel M.I.S., Lima V.L.A.G.d., Nascimento R.J.d. (2008). Capacidade antioxidante de frutas. Rev. Bras. De Ciências Farm..

[18] Angeloni C., Maraldi T., Milenkovic D., Vauzour D. (2015). Dietary polyphenols and their effects on cell biochemistry and pathophysiology 2014. Oxid. Med. Cell. Longev..

[19] Madani B., Mirshekari A., Yahia E.M., Golding J.B., Hajivand S., Dastjerdy A.M. (2021). Jamun (*Syzygium cumini* L. Skeels): A promising fruit for the future. Hortic. Rev..

[20] Singh B., Singh J.P., Kaur A., Singh N. (2018). Insights into the phenolic compounds present in jambolan (*Syzygium cumini*) along with their health-promoting effects. Int. J. Food Sci. Technol..

[21] Rodrigo R., Miranda A., Vergara L. (2011). Modulation of endogenous antioxidant system by wine polyphenols in human disease. Clin. Chim. Acta.

[22] Bensalem J., Dal-Pan A., Gillard E., Calon F., Pallet V. (2015). Protective effects of berry polyphenols against age-related cognitive impairment. Nutr. Aging.

[23] Hajipour S., Sarkaki A., Farbood Y., Eidi A., Mortazavi P., Valizadeh Z. (2016). Effect of gallic acid on dementia type of Alzheimer disease in rats: Electrophysiological and histological studies. Basic Clin. Neurosci..

[24] Klotz K., Weistenhöfer W., Neff F., Hartwig A., van Thriel C., Drexler H. (2017). The health effects of aluminum exposure. Dtsch. Ärzteblatt Int..

[25] Walton J.R. (2012). Evidence that Ingested Aluminum additives contained in processed foods and alum-treated drinking water are a major risk factor for Alzheimer’s Disease. Curr. Inorg. Chem. (Discontin.).

[26] Krewski D., Yokel R.A., Nieboer E., Borchelt D., Cohen J., Harry J., Kacew S., Lindsay J., Mahfouz A.M., Rondeau V. (2007). Human health risk assessment for aluminium, aluminium oxide, and aluminium hydroxide. J. Toxicol. Environ. Health Part B.

[27] Doungue H.T., Kengne A.P.N., Kuate D. (2018). Neuroprotective effect and antioxidant activity of Passiflora edulis fruit flavonoid fraction, aqueous extract, and juice in aluminum chloride-induced Alzheimer’s disease rats. Nutrire.

[28] Gulya K., Rakonczay Z., Kasa P. (1990). Cholinotoxic effects of aluminum in rat brain. J. Neurochem..

[29] Lukiw W.J., LeBlanc H.J., Carver L.A., McLachlan D.R., Bazan N.G. (1998). Run-on gene transcription in human neocortical nuclei: Inhibition by nanomolar aluminum and implications for neurodegenerative disease. J. Mol. Neurosci..

[30] Kawahara M., Kato-Negishi M. (2011). Link between aluminum and the pathogenesis of Alzheimer’s disease: The integration of the aluminum and amyloid cascade hypotheses. Int. J. Alzheimer’s Dis..

[31] Fish P.V., Steadman D., Bayle E.D., Whiting P. (2019). New approaches for the treatment of Alzheimer’s disease. Bioorg. Med. Chem. Lett..

[32] Dubois B., Hampel H., Feldman H.H., Scheltens P., Aisen P., Andrieu S., Bakardjian H., Benali H., Bertram L., Blennow K. (2016). Preclinical Alzheimer’s disease: Definition, natural history, and diagnostic criteria. Alzheimer’s Dement..

[33] Lu C.-T., Zhao Y.-Z., Wong H.L., Cai J., Peng L., Tian X.-Q. (2014). Current approaches to enhance CNS delivery of drugs across the brain barriers. Int. J. Nanomed..

[34] Kahkeshani N., Farzaei F., Fotouhi M., Alavi S.S., Bahramsoltani R., Naseri R., Momtaz S., Abbasabadi Z., Rahimi R., Farzaei M.H. (2019). Pharmacological effects of gallic acid in health and diseases: A mechanistic review. Iran. J. Basic Med. Sci..

[35] Ogunlade B., Adelakun S., Agie J. (2022). Nutritional supplementation of gallic acid ameliorates Alzheimer-type hippocampal neurodegeneration and cognitive impairment induced by aluminum chloride exposure in adult Wistar rats. Drug Chem. Toxicol..

[36] Reeves P.G., Nielsen F.H., Fahey G.C. (1993). AIN-93 Purified Diets for Laboratory Rodents: Final Report of the American Institute of Nutrition Ad Hoc Writing Committee on the Reformulation of the AIN-76A Rodent Diet.

[37] Singh J.P., Kaur A., Singh N., Nim L., Shevkani K., Kaur H., Arora D.S. (2016). In vitro antioxidant and antimicrobial properties of jambolan (*Syzygium cumini*) fruit polyphenols. LWT-Food Sci. Technol..

[38] Tarola A.M., Van de Velde F., Salvagni L., Preti R. (2013). Determination of phenolic compounds in strawberries (*Fragaria ananassa* Duch) by high performance liquid chromatography with diode array detection. Food Anal. Methods.

[39] Zaher M.F., Bendary M.A., Abd El-Aziz G.S., Ali A.S. (2020). Potential Protective Role of Thymoquinone on Experimentally-Induced Alzheimer Rats.

[40] Yassin E.M., El-Moslemany A.M. (2018). The Protective Effect of The Methanolic Extract of *Syzgium cumini* L Fruit on Kidney and Testes Tissue Damages Induced by Carbon Tetrachloride. Egypt. J. Food Sci..

[41] Carageorgiou H., Sideris A.C., Messari I., Liakou C.I., Tsakiris S. (2008). The effects of rivastigmine plus selegiline on brain acetylcholinesterase,(Na^+^, K^+^)-, Mg^2+^-ATPase activities, antioxidant status, and learning performance of aged rats. Neuropsychiatr. Dis. Treat..

[42] Velazquez R., Ferreira E., Knowles S., Fux C., Rodin A., Winslow W., Oddo S. (2019). Lifelong choline supplementation ameliorates Alzheimer’s disease pathology and associated cognitive deficits by attenuating microglia activation. Aging Cell.

[43] Thippeswamy A.H., Rafiq M., shastry Viswantha G.L., Kavya K.J., Anturlikar S.D., Patki P.S. (2013). Evaluation of *Bacopa monniera* for its synergistic activity with rivastigmine in reversing aluminum-induced memory loss and learning deficit in rats. J. Acupunct. Meridian Stud..

[44] Sasa S., Blank C.L. (1977). Determination of serotonin and dopamine in mouse brain tissue by high performance liquid chromatography with electrochemical detection. Anal. Chem..

[45] Cheney D.L., Lehmann J., Cosi C., Wood P.L. (1989). Determination of acetylcholine dynamics. Drugs as Tools in Neurotransmitter Research.

[46] Borish L., Rosenbaum R., Albury L., Clark S. (1989). Activation of neutrophils by recombinant interleukin 6. Cell. Immunol..

[47] Bergmeyer H., Herder M., Ref R. (1986). International federation of clinical chemistry (IFCC). J. Clin. Chem. Clin. Biochem..

[48] Nandi A., Chatterjee I. (1988). Assay of superoxide dismutase activity in animal tissues. J. Biosci..

[49] Uchiyama M., Mihara M. (1978). Determination of malonaldehyde precursor in tissues by thiobarbituric acid test. Anal. Biochem..

[50] Giustarini D., Rossi R., Milzani A., Dalle-Donne I. (2008). Nitrite and nitrate measurement by Griess reagent in human plasma: Evaluation of interferences and standardization. Methods Enzymol..

[51] Grafström G., Nittby H., Brun A., Malmgren L., Persson B.R., Salford L.G., Eberhardt J. (2008). Histopathological examinations of rat brains after long-term exposure to GSM-900 mobile phone radiation. Brain Res. Bull..

[52] Livak K.J., Schmittgen T.D. (2001). Analysis of relative gene expression data using real-time quantitative PCR and the 2− ΔΔCT method. Methods.

[53] Mohamed A.B., Mohamed A.Z., Aly S. (2020). Effect of Thymoquinone against Aluminum Chloride-Induced Alzheimer-Like Model in Rats: A Neurophysiological and Behavioral Study. Med. J. Cairo Univ..

[54] Ahmed R., Tariq M., Hussain M., Andleeb A., Masoud M.S., Ali I., Mraiche F., Hasan A. (2019). Phenolic contents-based assessment of therapeutic potential of Syzygium cumini leaves extract. PLoS ONE.

[55] Rajan M., Guedes T.J.F.L., Barbosa P.F., Araujoa H.C.S., Narain N. (2023). Development on chemical characteristics including the bioactive compounds and antioxidant activity during maturation of jambolan (*Syzygium cuminii* L.) fruit. J. Food Meas. Charact..

[56] Brusamarello B., da Silva J.C.C., de Morais Sousa K., Guarneri G.A. (2022). Bearing fault detection in three-phase induction motors using support vector machine and fiber Bragg grating. IEEE Sens. J..

[57] Naghizadeh B., Mansouri M. (2015). Protective effects of gallic acid against streptozotocin-induced oxidative damage in rat striatum. Drug Res..

[58] Prema A., Thenmozhi A.J., Manivasagam T., Essa M.M., Akbar M.D., Akbar M. (2016). Fenugreek seed powder nullified aluminium chloride induced memory loss, biochemical changes, Aβ burden and apoptosis via regulating Akt/GSK3β signaling pathway. PLoS ONE.

[59] Mohapatra D., Kanungo S., Pradhan S.P., Jena S., Prusty S.K., Sahu P.K. (2022). Captopril is more effective than Perindopril against aluminium chloride induced amyloidogenesis and AD like pathology. Heliyon.

[60] Zeisel S.H. (2000). Choline: An essential nutrient for humans. Nutrition.

[61] Pacelli C., Coluccia A., Grattagliano I., Cocco T., Petrosillo G., Paradies G., De Nitto E., Massaro A., Persichella M., Borracci P. (2010). Dietary choline deprivation impairs rat brain mitochondrial function and behavioral phenotype. J. Nutr..

[62] Jiang X., West A.A., Caudill M.A. (2014). Maternal choline supplementation: A nutritional approach for improving offspring health?. Trends Endocrinol. Metab..

[63] Pohanka M. (2012). Alpha7 nicotinic acetylcholine receptor is a target in pharmacology and toxicology. Int. J. Mol. Sci..

[64] Zotova E., Holmes C., Johnston D., Neal J.W., Nicoll J.A., Boche D. (2011). Microglial alterations in human Alzheimer’s disease following Aβ42 immunization. Neuropathol. Appl. Neurobiol..

[65] Srividhya R., Gayathri R., Kalaiselvi P. (2012). Impact of epigallo catechin-3-gallate on acetylcholine-acetylcholine esterase cycle in aged rat brain. Neurochem. Int..

[66] Cheruku S.P., Ramalingayya G.V., Chamallamudi M.R., Biswas S., Nandakumar K., Nampoothiri M., Gourishetti K., Kumar N. (2018). Catechin ameliorates doxorubicin-induced neuronal cytotoxicity in in vitro and episodic memory deficit in in vivo in Wistar rats. Cytotechnology.

[67] Jabir N.R., Khan F.R., Tabrez S. (2018). Cholinesterase targeting by polyphenols: A therapeutic approach for the treatment of Alzheimer’s disease. CNS Neurosci. Ther..

[68] Buraimoh A., Ojo S. (2014). Effects of Aluminium chloride exposure on the body weight of Wistar rats. Ann. Biol. Res..

[69] Balgoon M.J. (2019). Assessment of the protective effect of *Lepidium sativum* against aluminum-induced liver and kidney effects in albino rat. BioMed Res. Int..

[70] Lahouel Z., Kharoubi O., Boussadia A., Bekkouche Z., Aoues A. (2020). Effect of Aluminium and Aqueous extract of Rosmarinus officinalis on rat Brain: Impact on Neurobehavioral and Histological study. J. Drug Deliv. Ther..

[71] Bekhedda H., Menadi N., Demmouche A., Ghani A., Mai H. (2020). Histological study of the effects of aluminum chloride exposure on the brain of wistar rats female. J. Drug Deliv. Ther..

[72] Han S., Lemire J., Appanna V.P., Auger C., Castonguay Z., Appanna V.D. (2013). How aluminum, an intracellular ROS generator promotes hepatic and neurological diseases: The metabolic tale. Cell Biol. Toxicol..

[73] Rogge M.M. (2009). The role of impaired mitochondrial lipid oxidation in obesity. Biol. Res. Nurs..

[74] Kaushik V., Smith S.T., Mikobi E., Raji M.A. (2018). Acetylcholinesterase inhibitors: Beneficial effects on comorbidities in patients with Alzheimer’s disease. Am. J. Alzheimer’s Dis. Other Dement.^®^.

[75] Nordberg A., Ballard C., Bullock R., Darreh-Shori T., Somogyi M. (2013). A review of butyrylcholinesterase as a therapeutic target in the treatment of Alzheimer’s disease. Prim. Care Companion CNS Disord..

[76] Maya S., Prakash T., Madhu K.D., Goli D. (2016). Multifaceted effects of aluminium in neurodegenerative diseases: A review. Biomed. Pharmacother..

[77] Cohen E.L., Wurtman R.J. (1976). Brain acetylcholine: Control by dietary choline. Science.

[78] Kaizer R.R., Corrêa M.C., Spanevello R.M., Morsch V.M., Mazzanti C.M., Gonçalves J.F., Schetinger M.R. (2005). Acetylcholinesterase activation and enhanced lipid peroxidation after long-term exposure to low levels of aluminum on different mouse brain regions. J. Inorg. Biochem..

[79] Jasiecki J., Wasąg B. (2019). Butyrylcholinesterase protein ends in the pathogenesis of Alzheimer’s disease—Could BCHE genotyping be helpful in Alzheimer’s therapy?. Biomolecules.

[80] Hajialyani M., Hosein Farzaei M., Echeverría J., Nabavi S.M., Uriarte E., Sobarzo-Sánchez E. (2019). Hesperidin as a neuroprotective agent: A review of animal and clinical evidence. Molecules.

[81] Ju Y., Tam K.Y. (2022). Pathological mechanisms and therapeutic strategies for Alzheimer’s disease. Neural Regen. Res..

[82] Strandwitz P. (2018). Neurotransmitter modulation by the gut microbiota. Brain Res..

[83] Holland N., Robbins T.W., Rowe J.B. (2021). The role of noradrenaline in cognition and cognitive disorders. Brain.

[84] Butzlaff M., Ponimaskin E. (2016). The role of serotonin receptors in Alzheimer’s disease. Opera Med. Physiol..

[85] Ceyzériat K., Gloria Y., Tsartsalis S., Fossey C., Cailly T., Fabis F., Millet P., Tournier B.B. (2021). Alterations in dopamine system and in its connectivity with serotonin in a rat model of Alzheimer’s disease. Brain Commun..

[86] Pan X., Kaminga A.C., Wen S.W., Wu X., Acheampong K., Liu A. (2019). Dopamine and dopamine receptors in Alzheimer’s disease: A systematic review and network meta-analysis. Front. Aging Neurosci..

[87] Obafemi T.O., Owolabi O.V., Omiyale B.O., Afolabi B.A., Ojo O.A., Onasanya A., Adu I.A., Rotimi D. (2021). Combination of donepezil and gallic acid improves antioxidant status and cholinesterases activity in aluminum chloride-induced neurotoxicity in Wistar rats. Metab. Brain Dis..

[88] Aly H.F., Metwally F.M., Ahmed H.H. (2011). Neuroprotective effects of dehydroepiandrosterone (DHEA) in rat model of Alzheimer’s disease. Acta Biochim. Pol..

[89] Tsaluchidu S., Cocchi M., Tonello L., Puri B.K. (2008). Fatty acids and oxidative stress in psychiatric disorders. BMC Psychiatry.

[90] Nehru B., Anand P. (2005). Oxidative damage following chronic aluminium exposure in adult and pup rat brains. J. Trace Elem. Med. Biol..

[91] Abdel-Salam O.M., Hamdy S.M., Seadawy S.A.M., Galal A.F., Abouelfadl D.M., Atrees S.S. (2016). Effect of piracetam, vincamine, vinpocetine, and donepezil on oxidative stress and neurodegeneration induced by aluminum chloride in rats. Comp. Clin. Pathol..

[92] Oyetayo B.O., Abolaji A.O., Fasae K.D., Aderibigbe A. (2020). Ameliorative role of diets fortified with Curcumin in a *Drosophila melanogaster* model of aluminum chloride-induced neurotoxicity. J. Funct. Foods.

[93] Džoljić E., Grabatinić I., Kostić V. (2015). Why is nitric oxide important for our brain?. Funct. Neurol..

[94] Obafemi T.O., Olasehinde O.R., Olaoye O.A., Jaiyesimi K.F., Adewumi F.D., Adewale O.B., Afolabi B.A. (2020). Metformin/Donepezil combination modulates brain antioxidant status and hippocampal endoplasmic reticulum stress in type 2 diabetic rats. J. Diabetes Metab. Disord..

[95] Tseng P.S., Ande C., Moremen K.W., Crich D. (2023). Influence of side chain conformation on the activity of glycosidase inhibitors. Angew. Chem..

[96] Rajasekaran P., Ande C., Vankar Y.D. (2022). Synthesis of (5, 6 & 6, 6)-oxa-oxa annulated sugars as glycosidase inhibitors from 2-formyl galactal using iodocyclization as a key step. Arkivoc.

[97] Chennaiah A., Bhowmick S., Vankar Y.D. (2017). Conversion of glycals into vicinal-1, 2-diazides and 1, 2-(or 2, 1)-azidoacetates using hypervalent iodine reagents and Me_3_SiN_3_. Application in the synthesis of N-glycopeptides, pseudo-trisaccharides and an iminosugar. RSC Adv..

[98] Parekh K.D., Dash R.P., Pandya A.N., Vasu K.K., Nivsarkar M. (2013). Implication of novel bis-imidazopyridines for management of Alzheimer’s disease and establishment of its role on protein phosphatase 2A activity in brain. J. Pharm. Pharmacol..

[99] Petrovic S., Arsic A., Ristic-Medic D., Cvetkovic Z., Vucic V. (2020). Lipid peroxidation and antioxidant supplementation in neurodegenerative diseases: A review of human studies. Antioxidants.

[100] Li Q., Wang J., Li Y., Xu X. (2018). Neuroprotective effects of salidroside administration in a mouse model of Alzheimer’s disease. Mol. Med. Rep..

[101] Kong D., Yan Y., He X.-Y., Yang H., Liang B., Wang J., He Y., Ding Y., Yu H. (2019). Effects of resveratrol on the mechanisms of antioxidants and estrogen in Alzheimer’s disease. BioMed Res. Int..

[102] Nabila M.R. (2012). Effect of physalis and choline on lipid profile and antioxidant activity in hepatic toxicity rats. Aust. J. Basic Appl. Sci..

[103] Shi B., Hu X., Jin M., Xia M., Zhao M., Jiao L., Sun P., Zhou Q. (2021). Dietary choline improves growth performance, antioxidant ability and reduces lipid metabolites in practical diet for juvenile Pacific white shrimp, *Litopenaeus vannamei*. Aquac. Nutr..

[104] Chan P., Cheng J.-T., Tsai J.-C., Lien G.-S., Chen F.-C., Kao P.-F., Liu J.-C., Chen Y.-J., Hsieh M.-H. (2002). Effect of catechin on the activity and gene expression of superoxide dismutase in cultured rat brain astrocytes. Neurosci. Lett..

[105] Yeh C.-T., Yen G.-C. (2003). Effects of phenolic acids on human phenolsulfotransferases in relation to their antioxidant activity. J. Agric. Food Chem..

[106] Song F., Li H., Sun J., Wang S. (2013). Protective effects of cinnamic acid and cinnamic aldehyde on isoproterenol-induced acute myocardial ischemia in rats. J. Ethnopharmacol..

[107] Liu L., Liu Y., Zhao J., Xing X., Zhang C., Meng H. (2020). Neuroprotective effects of D-(-)-quinic acid on aluminum chloride-induced dementia in rats. Evid.-Based Complement. Altern. Med..

[108] Shunan D., Yu M., Guan H., Zhou Y. (2021). Neuroprotective effect of Betalain against AlCl3-induced Alzheimer’s disease in Sprague Dawley Rats via putative modulation of oxidative stress and nuclear factor kappa B (NF-κB) signaling pathway. Biomed. Pharmacother..

[109] Kim J., Wie M.-B., Ahn M., Tanaka A., Matsuda H., Shin T. (2019). Benefits of hesperidin in central nervous system disorders: A review. Anat. Cell Biol..

[110] Cheng X.-J., Gu J.-X., Pang Y.-P., Liu J., Xu T., Li X.-R., Hua Y.-Z., Newell K.A., Huang X.-F., Yu Y. (2019). Tacrine–hydrogen sulfide donor hybrid ameliorates cognitive impairment in the aluminum chloride mouse model of Alzheimer’s disease. ACS Chem. Neurosci..

[111] Raj M. H., Ghosh D., Banerjee R., Salimath B.P. (2017). Suppression of VEGF-induced angiogenesis and tumor growth by *Eugenia jambolana*, *Musa paradisiaca*, and *Coccinia indica* extracts. Pharm. Biol..

[112] Wang W.-Y., Tan M.-S., Yu J.-T., Tan L. (2015). Role of pro-inflammatory cytokines released from microglia in Alzheimer’s disease. Ann. Transl. Med..

[113] Su F., Bai F., Zhang Z. (2016). Inflammatory cytokines and Alzheimer’s disease: A review from the perspective of genetic polymorphisms. Neurosci. Bull..

[114] Kinney J.W., Bemiller S.M., Murtishaw A.S., Leisgang A.M., Salazar A.M., Lamb B.T. (2018). Inflammation as a central mechanism in Alzheimer’s disease. Alzheimer’s Dement. Transl. Res. Clin. Interv..

[115] Milnerowicz H., Ściskalska M., Dul M. (2015). Pro-inflammatory effects of metals in persons and animals exposed to tobacco smoke. J. Trace Elem. Med. Biol..

[116] Lee S., Kim H.-B., Hwang E.-S., Kim E.-S., Kim S.-S., Jeon T.-D., Song M.-C., Lee J.-S., Chung M.-C., Maeng S. (2018). Antidepressant-like effects of p-coumaric acid on LPS-induced depressive and inflammatory changes in rats. Exp. Neurobiol..

[117] Mellott T.J., Huleatt O.M., Shade B.N., Pender S.M., Liu Y.B., Slack B.E., Blusztajn J.K. (2017). Perinatal choline supplementation reduces amyloidosis and increases choline acetyltransferase expression in the hippocampus of the APPswePS1dE9 Alzheimer’s disease model mice. PLoS ONE.

[118] Zeisel S.H. (2017). Choline, other methyl-donors and epigenetics. Nutrients.

[119] Liaquat L., Sadir S., Batool Z., Tabassum S., Shahzad S., Afzal A., Haider S. (2019). Acute aluminum chloride toxicity revisited: Study on DNA damage and histopathological, biochemical and neurochemical alterations in rat brain. Life Sci..

[120] Al-Hazmi M.A., Rawi S.M., Hamza R.Z. (2021). Biochemical, histological, and neuro-physiological effects of long-term aluminum chloride exposure in rats. Metab. Brain Dis..

[121] Kumaran D., Hassabis D., McClelland J.L. (2016). What learning systems do intelligent agents need? Complementary learning systems theory updated. Trends Cogn. Sci..

[122] Buraimoh A., Ojo S., Hambolu J., Adebisi S. (2011). Effects of oral administration of aluminium chloride on the histology of the hippocampus of wistar rats. Curr. Res. J. Biol. Sci..

[123] Buraimoh A., Ojo S. (2012). Effects of aluminium chloride exposure on the histology of the stomach of wistar rats. Int. J. Pharm. Bio Sci..

[124] Ekundayo B.E., Obafemi T.O., Afolabi B.A., Adewale O.B., Onasanya A., Osukoya O.A., Falode J.A., Akintayo C., Adu I.A. (2022). Gallic acid and hesperidin elevate neurotransmitters level and protect against oxidative stress, inflammation and apoptosis in aluminum chloride-induced Alzheimer’s disease in rats. Pharmacol. Res.-Mod. Chin. Med..

[125] Zou J., Cai P.-S., Xiong C.-M., Ruan J.-L. (2016). Neuroprotective effect of peptides extracted from walnut (Juglans Sigilata Dode) proteins on Aβ25-35-induced memory impairment in mice. J. Huazhong Univ. Sci. Technol. [Med. Sci.].

[126] Kumar E., Mastan S., Reddy K.R., Reddy G.A., Raghunandan N., Chaitanya G. (2008). Anti-arthritic property of the methanolic extract of *Syzygium cumini* seeds. Int. J. Integr. Biol..

[127] Liu L., Lu Y., Bi X., Xu M., Yu X., Xue R., He X., Zang W. (2017). Choline ameliorates cardiovascular damage by improving vagal activity and inhibiting the inflammatory response in spontaneously hypertensive rats. Sci. Rep..

[128] McMaster W.G., Kirabo A., Madhur M.S., Harrison D.G. (2015). Inflammation, immunity, and hypertensive end-organ damage. Circ. Res..

