# Supplementary material for: Assessment of Anti-Alzheimer Pursuit of Jambolan Fruit Extract and/or Choline against AlCl3 Toxicity in Rats
Source: Toxics. 2023 Jun 6;11(6):509. doi: 10.3390/toxics11060509 (PMC10305215; doi:10.3390/toxics11060509)
Supplement: Supplementary file 1 [file toxics-11-00509-s001.zip › toxics-2394906-supplementary.pdf]

Z. abdelsalam
65mg/1.5ml

===

Injection Date : 8/29/2022 7:29:33 PM
Sample Name : jambozia Location : Vial 51
Acq. Operator : 12
Acq. Instrument : Instrument 1 Inj Volume : 25 µl
Method : C:\HPCHEM\1\METHODS\PHENOLS2.M
Last changed : 8/29/2022 7:28:07 PM by 12
(modified after loading)

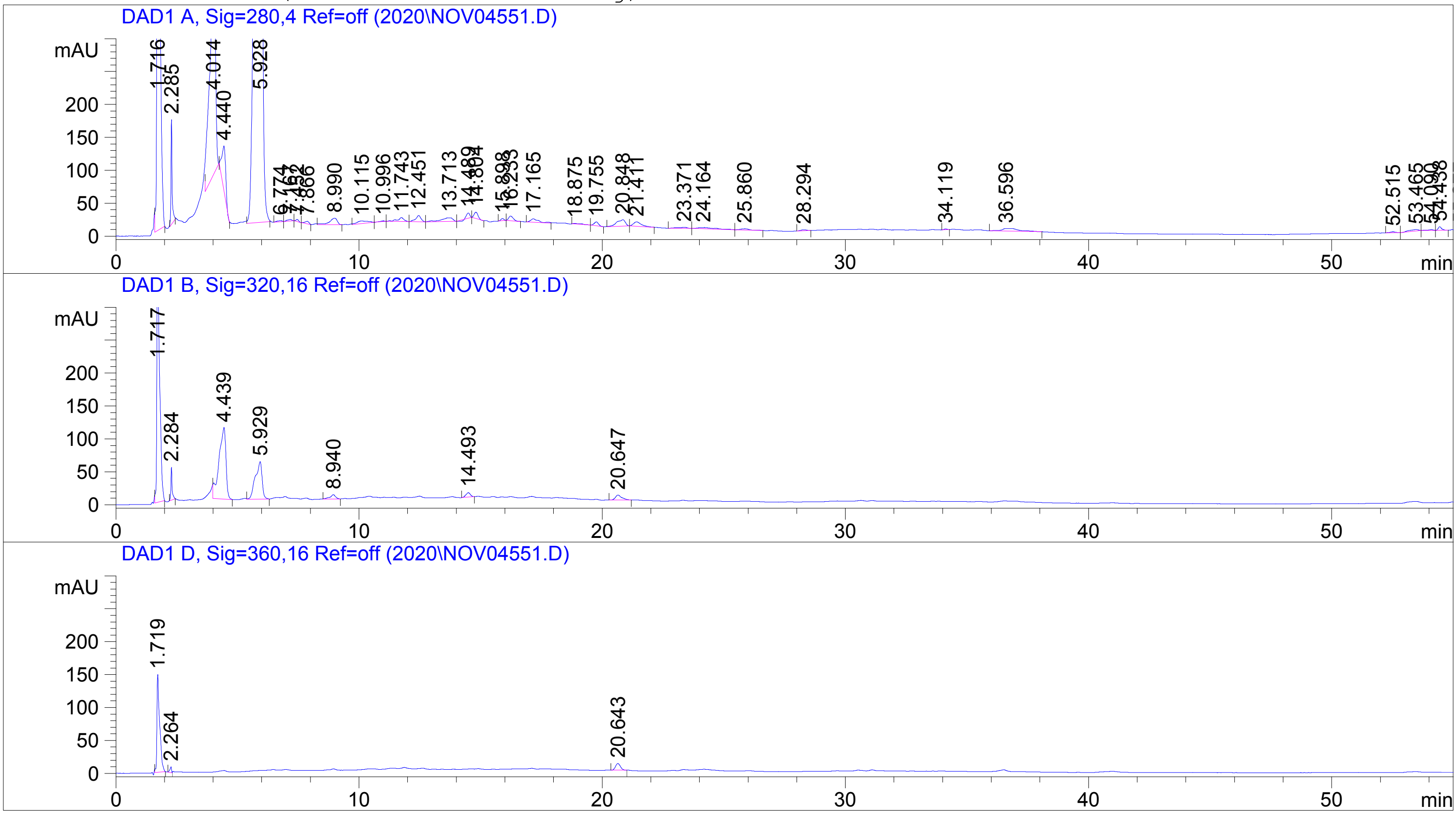

===

Area Percent Report

===

Sorted By : Signal
Multiplier : 1.0000
Dilution : 1.0000
Use Multiplier & Dilution Factor with ISTDs

Signal 1: DAD1 A, Sig=280,4 Ref=off

Peak #	RetTime [min]	Type	Width [min]	Area [mAU*s]	Height [mAU]	Area %
1	1.716	BBA	0.1003	6861.94336	927.29596	12.0603
2	2.285	PBA	0.0519	543.70203	158.99638	0.9556
3	4.014	BB	0.2109	5484.32959	365.78336	9.6390
4	4.440	VBA	0.1603	717.07520	67.31033	1.2603
5	5.928	PBA	0.2672	2.02850e4	1060.14404	35.6520
6	6.774	PB	0.1932	24.20934	1.79629	0.0425
7	7.167	VB	0.2080	34.79990	2.41613	0.0612
8	7.452	VBA	0.1294	25.45391	3.17896	0.0447
9	7.866	BBA	0.1733	42.61522	3.72965	0.0749
10	8.990	BBA	0.3483	224.67941	9.76897	0.3949
11	10.115	PB	0.4607	131.08319	4.01711	0.2304
12	10.996	VB	0.1881	17.02694	1.27228	0.0299
13	11.743	VB	0.2675	119.20752	6.11307	0.2095
14	12.451	VB	0.2277	144.74051	9.39816	0.2544
15	13.713	VB	0.5075	189.65312	5.37689	0.3333
16	14.489	VB	0.1405	60.52457	7.98264	0.1064
17	14.804	VBA	0.1826	107.00478	9.55721	0.1881

Peak #	RetTime [min]	Type	Width [min]	Area [mAU*s]	Height [mAU]	Area %
18	15.898	PB	0.1433	27.47092	3.10374	0.0483
19	16.233	VBA	0.2024	89.89925	6.97476	0.1580
20	17.165	PBA	0.3144	121.80498	5.25579	0.2141
21	18.875	PB	0.3451	29.39196	1.15413	0.0517
22	19.755	VBA	0.1918	72.97198	5.92058	0.1283
23	20.848	PB	0.3338	233.85417	9.54711	0.4110
24	21.411	VBA	0.3365	150.23920	6.62641	0.2641
25	23.371	PB	0.3663	45.62923	1.71567	0.0802
26	24.164	VB	0.7309	90.77269	1.85507	0.1595
27	25.860	VBA	0.3745	48.88525	1.93646	0.0859
28	28.294	PBA	0.2588	28.81634	1.72556	0.0506
29	34.119	PBA	0.1451	11.49971	1.27670	0.0202
30	36.596	PBA	0.6554	201.61954	3.82211	0.3544
31	52.515	PB	0.2395	23.39412	1.48762	0.0411
32	53.465	VB	0.4130	68.64340	2.34825	0.1206
33	54.090	VB	0.2734	22.66804	1.21640	0.0398
34	54.438	VBA	0.1578	53.92165	5.34110	0.0948
35	55.277	BBA	0.1884	22.79411	1.79252	0.0401
36	56.336	BB	0.2280	20.38576	1.21368	0.0358
37	56.832	VBA	0.1449	12.15642	1.30203	0.0214
38	58.717	PBA	0.0597	41.73817	11.15557	0.0734
39	59.320	PBA	0.0935	2340.53638	361.77420	4.1136
40	59.791	PBA	0.1444	1.79128e4	1928.31763	31.4828
41	60.773	BB	0.2238	44.52031	3.09468	0.0782
42	62.047	VBA	0.1792	167.65778	13.66077	0.2947

Totals : 5.68971e4 5027.75599

Results obtained with enhanced integrator!

Signal 2: DAD1 B, Sig=320,16 Ref=off

Peak #	RetTime [min]	Type	Width [min]	Area [mAU*s]	Height [mAU]	Area %
1	1.717	BBA	0.0983	3065.87061	424.28552	34.0193
2	2.284	PBA	0.0542	179.58778	49.67238	1.9927
3	4.439	BBA	0.2734	2169.90576	108.45674	24.0776
4	5.929	BBA	0.2663	1104.71802	56.95844	12.2581
5	8.940	BBA	0.2345	107.58514	6.59008	1.1938
6	14.493	PBA	0.2046	86.60702	6.61957	0.9610
7	20.647	BBA	0.2693	142.33298	7.64121	1.5793
8	59.114	PB	0.0746	30.65472	6.31337	0.3401
9	59.322	VBA	0.1036	1933.99695	269.88635	21.4599
10	60.155	PB	0.0712	41.22239	9.38686	0.4574
11	60.340	VB	0.0751	67.48535	14.29713	0.7488
12	60.505	VBA	0.0704	61.38551	14.20889	0.6811
13	62.077	BB	0.0685	20.80050	5.00212	0.2308

Totals : 9012.15271 979.31866

Results obtained with enhanced integrator!

Signal 3: DAD1 D, Sig=360,16 Ref=off

Peak #	RetTime [min]	Type	Width [min]	Area [mAU*s]	Height [mAU]	Area %
1	1.719	BBA	0.1066	1153.15784	148.48129	61.0249
2	2.264	BBA	0.0611	37.54815	8.93021	1.9870
3	20.643	PBA	0.2188	141.80185	10.15965	7.5041
4	59.322	PBA	0.0971	513.14935	77.61026	27.1558
5	60.503	BBA	0.1132	43.99489	5.50652	2.3282

Totals : 1889.65208 250.68792

Results obtained with enhanced integrator!

==

*** End of Report ***

phenol

===

Injection Date : 8/29/2022 6:05:59 PM
Sample Name : std Location : Vial 1
Acq. Operator : 12
Acq. Instrument : Instrument 1 Inj Volume : 10 µl
Acq. Method : C:\HPCHEM\1\METHODS\PHENOLS2.M
Last changed : 8/29/2022 6:04:50 PM by 12
(modified after loading)
Analysis Method : C:\HPCHEM\1\METHODS\PHENOLS2.M
Last changed : 8/29/2022 8:54:13 PM by 12
(modified after loading)

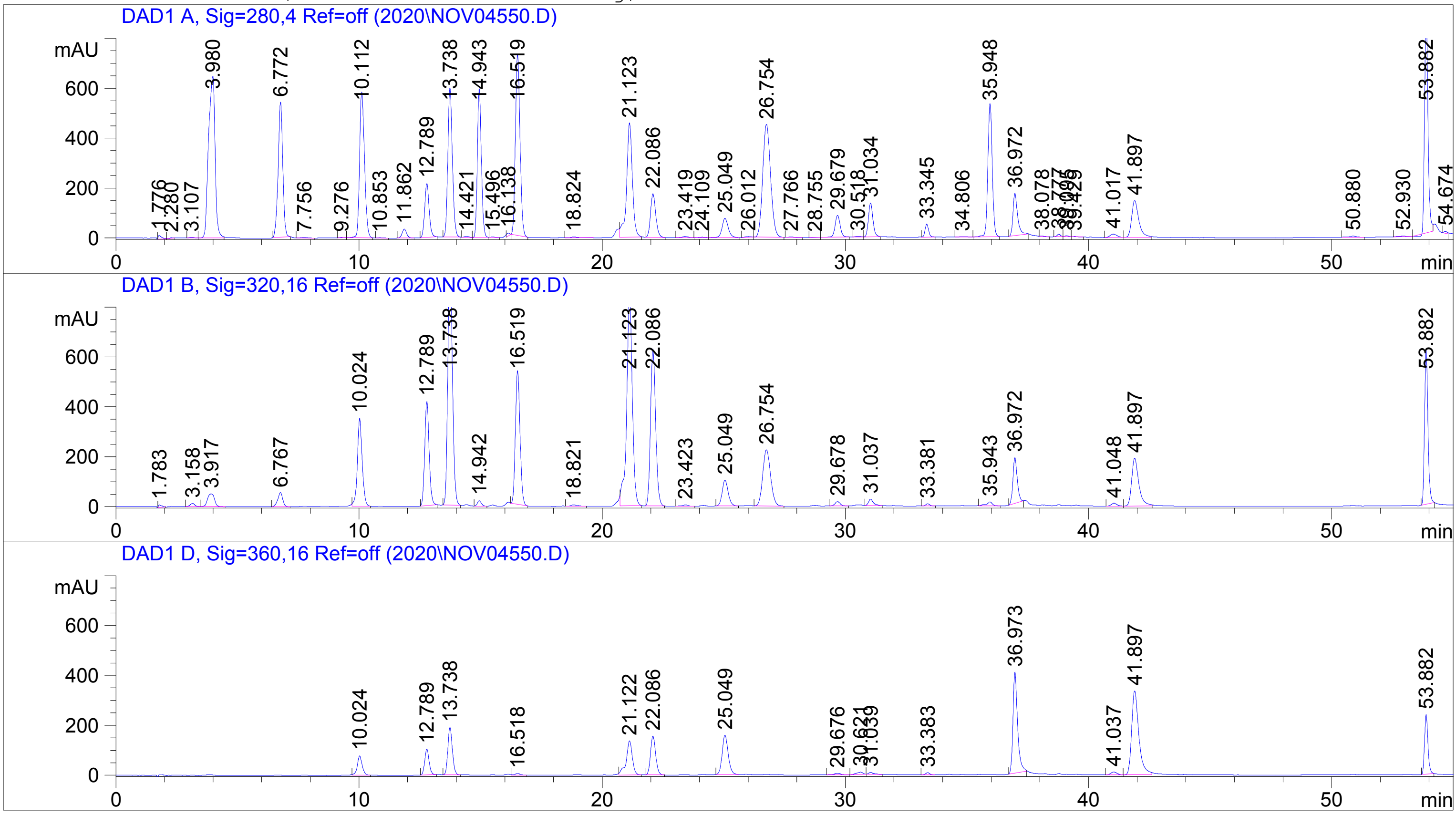

===

Area Percent Report

===

Sorted By : Signal
Multiplier : 1.0000
Dilution : 1.0000
Use Multiplier & Dilution Factor with ISTDs

Signal 1: DAD1 A, Sig=280,4 Ref=off

Peak #	RetTime [min]	Type	Width [min]	Area [mAU*s]	Height [mAU]	Area %
1	1.776	PB	0.1304	145.20306	15.34984	0.1178
2	2.280	VBA	0.1206	20.81437	2.51428	0.0169
3	3.107	PB	0.1853	29.30627	2.56464	0.0238
4	3.980	PBA	0.3083	1.20159e4	648.09680	9.7471
5	6.772	BBA	0.2021	7147.78516	541.00671	5.7982
6	7.756	BBA	0.2485	47.41416	2.93516	0.0385
7	9.276	PB	0.1950	15.52910	1.30332	0.0126
8	10.112	VBA	0.2162	8207.52930	583.12683	6.6578
9	10.853	PBA	0.2039	15.58479	1.26380	0.0126
10	11.862	BBA	0.2016	464.19873	36.20041	0.3765
11	12.789	PBA	0.1963	2748.69336	216.16930	2.2297
12	13.738	BBA	0.2036	7736.05859	595.31335	6.2754
13	14.421	BB	0.1879	52.50232	4.37975	0.0426
14	14.943	VBA	0.1769	6788.07373	595.89319	5.5064
15	15.496	PBA	0.1553	23.23472	2.35045	0.0188

Peak #	RetTime [min]	Type	Width [min]	Area [mAU*s]	Height [mAU]	Area %
16	16.138	PB	0.1464	96.99231	11.49631	0.0787
17	16.519	VBA	0.2059	9586.00781	726.70435	7.7760
18	18.824	BBA	0.2975	68.96020	3.33174	0.0559
19	21.123	BBA	0.2497	7596.95361	457.57028	6.1625
20	22.086	BBA	0.2308	2612.27051	174.38321	2.1190
21	23.419	PB	0.2597	77.00349	4.58946	0.0625
22	24.109	VB	0.2568	27.50188	1.81492	0.0223
23	25.049	VBA	0.2704	1355.31458	76.60731	1.0994
24	26.012	PB	0.2495	46.66495	3.06668	0.0379
25	26.754	VBA	0.3509	1.01797e4	451.75534	8.2576
26	27.766	BBA	0.2494	38.27962	2.30903	0.0311
27	28.755	PB	0.2243	20.36964	1.41218	0.0165
28	29.679	VBA	0.2296	1308.08447	87.90252	1.0611
29	30.518	PB	0.2977	42.49326	2.45740	0.0345
30	31.034	VBA	0.2000	1757.38672	134.92480	1.4256
31	33.345	BBA	0.1544	532.08350	52.44175	0.4316
32	34.806	PB	0.2605	26.67685	1.49287	0.0216
33	35.948	VBA	0.1947	6711.89941	533.85681	5.4446
34	36.972	PBA	0.1977	2234.01904	169.56470	1.8122
35	38.078	BBA	0.1859	19.05911	1.44445	0.0155
36	38.777	PB	0.1531	91.47002	9.43530	0.0742
37	39.095	VB	0.1546	29.88517	3.15110	0.0242
38	39.429	VBA	0.1830	16.62256	1.35598	0.0135
39	41.017	PB	0.3063	256.63104	13.46881	0.2082
40	41.897	VBA	0.3083	2988.93872	147.59384	2.4246
41	50.880	BBA	0.2843	92.62884	4.73457	0.0751
42	52.930	PB	0.3570	63.46002	2.39623	0.0515
43	53.882	VBA	0.1527	8556.11719	885.80542	6.9406
44	54.674	BBA	0.1361	67.69601	7.58861	0.0549
45	55.423	VB	0.1070	8.41187	1.38140	6.824e-3
46	56.848	VBA	0.5464	87.56845	2.01730	0.0710
47	58.212	PB	0.1444	160.16315	17.89057	0.1299
48	58.688	PBA	0.0607	49.88667	13.01328	0.0405
49	59.283	PBA	0.0923	2793.92017	451.10129	2.2664
50	59.745	PBA	0.1488	1.77179e4	1832.54846	14.3724
51	61.300	VBA	0.4833	336.35559	8.46968	0.2728
52	61.927	PBA	0.1439	163.64844	17.37870	0.1327

Totals : 1.23277e5 9566.92447

Results obtained with enhanced integrator!

Signal 2: DAD1 B, Sig=320,16 Ref=off

Peak #	RetTime [min]	Type	Width [min]	Area [mAU*s]	Height [mAU]	Area %
1	1.783	PBA	0.1257	99.52183	10.77702	0.1266
2	3.158	BBA	0.1907	157.13232	12.84486	0.1999
3	3.917	VBA	0.3262	998.45508	50.59586	1.2702
4	6.767	BBA	0.2150	810.00751	56.57182	1.0305
5	10.024	BBA	0.2046	4854.06299	352.60684	6.1751
6	12.789	PBA	0.1958	5283.75146	416.98819	6.7218
7	13.738	BBA	0.1995	1.31632e4	1013.37085	16.7457
8	14.942	PBA	0.1750	243.54747	21.68786	0.3098
9	16.519	PBA	0.2058	7057.86572	535.47009	8.9787
10	18.821	BBA	0.2646	106.03281	5.81958	0.1349
11	21.123	BBA	0.2457	1.36823e4	841.41803	17.4061
12	22.086	BBA	0.2279	9254.64746	628.14276	11.7734
13	23.423	BBA	0.2471	89.39497	5.45681	0.1137
14	25.049	PBA	0.2697	1793.90637	103.75544	2.2821
15	26.754	BBA	0.3508	5062.41064	224.75359	6.4402
16	29.678	BBA	0.2320	263.94879	17.49628	0.3358
17	31.037	PBA	0.2147	365.80841	25.59489	0.4654
18	33.381	BBA	0.1800	101.13263	8.67873	0.1287
19	35.943	BBA	0.2546	282.29605	15.94406	0.3591
20	36.972	PBA	0.1881	2263.90015	183.24100	2.8800
21	41.048	PB	0.2423	182.36859	11.66902	0.2320
22	41.897	VBA	0.3086	3883.83545	191.48112	4.9409
23	53.882	PBA	0.1492	6008.62988	619.07715	7.6439
24	59.075	PB	0.0703	38.71469	8.62852	0.0493
25	59.283	VBA	0.0965	2199.76562	335.60617	2.7985

Peak #	RetTime [min]	Type	Width [min]	Area [mAU*s]	Height [mAU]	Area %
26	60.451	PBA	0.0732	35.92463	7.87847	0.0457
27	62.404	BBA	0.3831	323.88855	10.40805	0.4120

Totals : 7.86064e4 5715.96306

Results obtained with enhanced integrator!

Signal 3: DAD1 D, Sig=360,16 Ref=off

Peak #	RetTime [min]	Type	Width [min]	Area [mAU*s]	Height [mAU]	Area %
1	10.024	BBA	0.2061	1079.79700	77.71843	3.7936
2	12.789	PBA	0.1946	1287.66370	102.50054	4.5238
3	13.738	PBA	0.1993	2449.56958	188.85277	8.6059
4	16.518	BBA	0.2295	96.30482	6.32858	0.3383
5	21.122	BBA	0.2606	2398.13721	136.75647	8.4252
6	22.086	PBA	0.2274	2296.56396	156.30453	8.0683
7	25.049	BBA	0.2664	2734.06470	157.62489	9.6054
8	29.676	BBA	0.2785	122.99583	6.56717	0.4321
9	30.621	PB	0.2700	162.29652	8.84777	0.5702
10	31.039	VBA	0.2210	110.37918	7.44563	0.3878
11	33.383	BBA	0.1776	96.01796	8.38738	0.3373
12	36.973	PBA	0.1974	5315.88428	404.26114	18.6759
13	41.037	PB	0.2732	194.96971	11.30743	0.6850
14	41.897	VBA	0.3058	6851.20752	336.07614	24.0698
15	53.882	PBA	0.1491	2310.08667	238.35675	8.1158
16	59.283	PBA	0.0918	595.98303	96.86105	2.0938
17	62.478	BBA	0.4865	361.98962	10.39005	1.2717

Totals : 2.84639e4 1954.58672

Results obtained with enhanced integrator!

*** End of Report ***

Table 1S. Primers for gene expression by RT-PCR

Gene	Direction	Primer sequence	Accession number
iKB- α	F:	CGGGAGAATGACGTGAAGGT	NM_053355
	R:	AGCAGCAGCCGTACCATATC	
NF-κB	F:	TTCAACATGGCAGACGACGA	NM_001415012
	R:	GGTATGGGCCATCTGCTGTT	
iNOS	F:	TGGGTGAAAGCGGTGTTCTT	S71597
	R:	TAGCGCTTCCGACTTCCTTG	
GAPDH	F:	TGGGTGAAAGCGGTGTTCTT	NM_017008
	R:	TAGCGCTTCCGACTTCCTTG	

iKB- α, inhibitor of nuclear factor kappa B kinase ,
iNOS,Inducible nitric oxide synthase
NF-κB. nuclear factor kappa b.
GAPDH, glyceraldehyde-3-phosphate dehydrogenase. SOD, superoxide dismutase.
